# Design of New Antibacterial Enhancers Based on AcrB’s Structure and the Evaluation of Their Antibacterial Enhancement Activity

**DOI:** 10.3390/ijms17111934

**Published:** 2016-11-18

**Authors:** Yi Song, Rongxin Qin, Xichun Pan, Qin Ouyang, Tianyu Liu, Zhaoxia Zhai, Yingchun Chen, Bin Li, Hong Zhou

**Affiliations:** 1 Department of Pharmacology, College of Pharmacy, The Third Military Medical University, Chongqing 400038, China; songyi19890126@163.com (Y.S.); michel_0415@163.com (R.Q.); xichunpan@163.com (X.P.); zzzhaoxia@sina.com (Z.Z.); libin6033@sina.com (B.L.); 2 Department of Medicinal Chemistry, College of Pharmacy, The Third Military Medical University, Chongqing 400038, China; ouyangq@tmmu.edu.cn (Q.O.); liutianyu@tmmu.edu.cn (T.L.); ycchen@scu.edu.cn (Y.C.)

**Keywords:** antibacterial enhancer, AcrB inhibitor, DHA27, molecular mechanism

## Abstract

Previously, artesunate (AS) and dihydroartemisinine 7 (DHA7) were found to have antibacterial enhancement activity against *Escherichia coli* via inhibition of the efflux pump AcrB. However, they were only effective against *E. coli* standard strains. This study aimed to develop effective antibacterial enhancers based on the previous work. Our results demonstrate that 86 new antibacterial enhancers were designed via 3D-SAR and molecular docking. Among them, DHA27 had the best antibacterial enhancement activity. It could potentiate the antibacterial effects of ampicillin against not only *E. coli* standard strain but also clinical strains, and of β-lactam antibiotics, not non-β-lactamantibiotics. DHA27 could increase the accumulation of daunomycin and nile red within *E. coli* ATCC 35218, but did not increase the bacterial membrane permeability. DHA27 reduced *acrB*’s mRNA expression of *E. coli* ATCC 35218 in a dose-dependent manner, and its antibacterial enhancement activity is related to the degree of *acrB* mRNA expression in *E. coli* clinical strains. The polypeptides from AcrB were obtained via molecular docking assay; the pre-incubated polypeptides could inhibit the activity of DHA27. Importantly, DHA27 had no cytotoxicity on cell proliferation. In conclusion, among newly designed antibacterial enhancers, DHA27 had favorable physical and pharmacological properties with no significant cytotoxicity at effective concentrations, and might serve as a potential efflux pump inhibitor in the future.

## 1. Introduction

Multidrug resistance (MDR) in gram-negative pathogens, including *Escherichia coli* (*E. coli*), *Pseudomonas aeruginosa*, and *Klebsiellap neumoniae*, has become a major concern in public health [1]. The resistant mechanism of MDR gram-negative strains is closely related to efflux pumps of the resistance nodulation division (RND) family [2]. The major RND efflux system in *E. coli* is AcrAB-TolC, which consists of a cell membrane transporter (AcrB), an inner membrane channel (TolC) and an adaptor protein (AcrA) [3]. Substrates of AcrAB-TolC get into the periplasmic space through porin or by diffusion through the lipid bilayer, where they are captured by AcrB. The AcrB transporter extrudes the compounds into the TolC channel and to the exterior using the proton motive force [4]. Therefore, the inhibition of AcrB function can inhibit the functions of AcrAB-TolC efflux pump. Recently, efflux pump inhibitors (EPIs)/AcrB inhibitors have emerged as a hot topic and have attracted the interest of many researchers.

Biochemical studies have verified that EPIs can be used as synergism therapies with antibiotics to improve the antibacterial potency at low antibiotic concentrations to reduce the emergence of resistance [5,6,7]. So far, several potent AcrB inhibitors including phenylalanylarginine-β-Naphthylamide (PAβN), 1-(1-naphthylmethyl)-piperazine (NMP), D13-9001, and pyranopyridines (MBX2319) have been described in the literature [8]. However, none of them is being used clinically.

Previously, it was found that artesunate (AS), an antimalarial agent, had no direct antibacterial activity itself, but it could significantly enhance the antibacterial effects of β-lactam antibiotics against *E. coli* ATCC 35218 in vitro, which might be associated with the efflux pump AcrB [9]. Based on the results of molecular docking experiments, a new antibiotic enhancer named as dihydroartemisinine 7 (DHA7) was developed by removing the carbonyl group and replacing the carboxyl group with imidazole in the succinate tail of AS. DHA7 has better antibacterial enhancement activity of β-lactam antibiotics than AS does [10]. This experiment proved that the strategy of changing the chemical structure of the succinate tail of AS to obtain a better active compound targeting AcrB was feasible. However, AS and DHA7 were effective only against standard strains. Most of *E. coli* clinical strains were not susceptible to them. Additionally, the aqueous solubility of these compounds was so poor that they could not be developed for clinical application. This study reported the whole process from design, synthesis, to evaluation of a new antibacterial enhancer DHA27 with better activity than those of AS and DHA7, and its possible molecular mechanism.

## 2. Results

### 2.1. Eighty-Six New Compounds Are Designed Based on Structure–Activity Relationship Analysis of 18 Artemisinin Derivatives from a Previous Work

**Figure 1:**
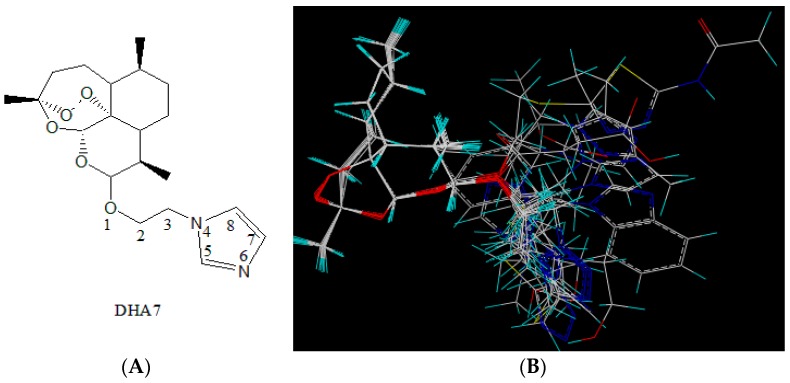

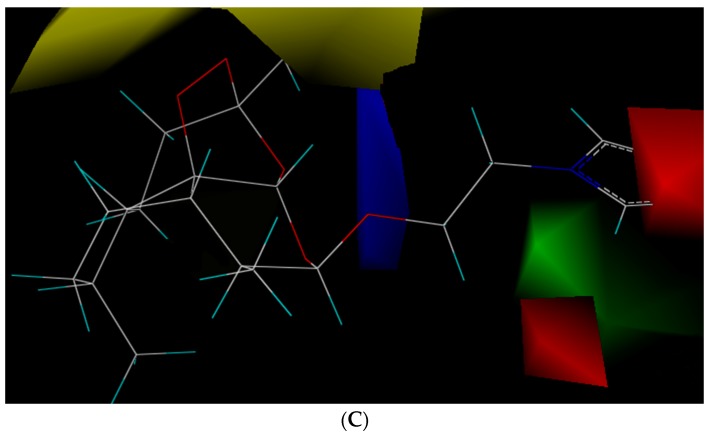
Three-dimensional structure–activity relationship (3D-SAR) analysis. (**A**) DHA7 as a molecular template; (**B**) A total of 18 compounds were used for database alignment. The molecular energy minimization of each compound was calculated in the compute mode; (**C**) Comparative molecular field analysis results (including steric and electrostatic fields).

Eighteen artemisinin (ART) derivatives have been derived from a previous work so far [10]. DHA7 was selected as the molecular template (Figure 1A). This compound was chosen as it was the most potent enhancer among 18 compounds. Within the best fit conformation for alignment, the left part of the figure shows the nucleus structure of ART and the right part shows the side chains of derivatives (Figure 1B).

The statistical parameters of CoMFA met the requirement and are summarized in Table S1. The results showed that (1) if steric hindrance increased in yellow part, bioactivity would decrease (yellow part); (2) if there were big steric hindrance groups on C-3 in side chains (green part), antibacterial enhancement activity would increase; (3) if there were electron-withdrawing groups at C-5 and C-7 (red part) and the electron-donating groups at O-1 (blue part), antibacterial enhancement activity would increase (Figure 1C). Therefore, 86 new compounds were designed by computer according to the aforementioned findings.

### 2.2. Twenty-Two Compounds Combined with the Active Binding Pocket from AcrB Are Selected Based on Molecular Docking

**Figure 2:**
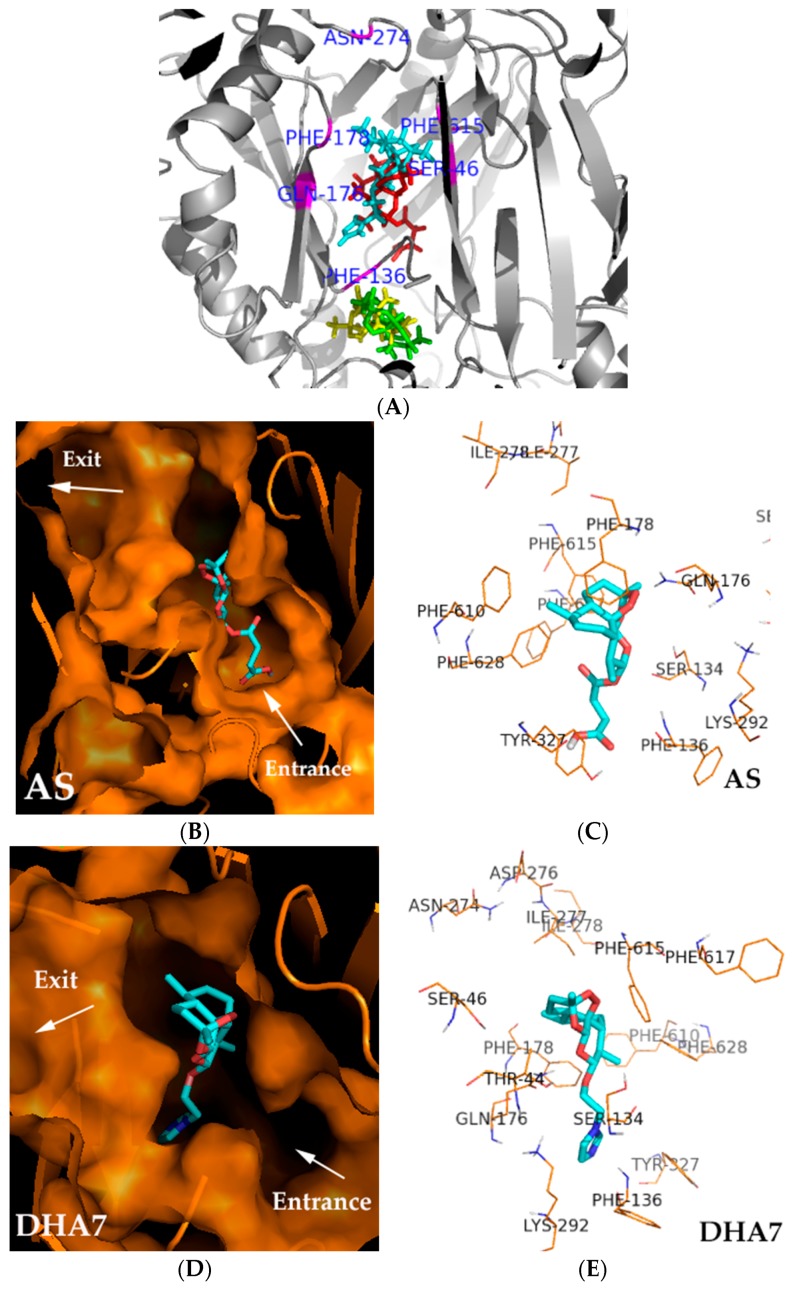
Molecule docking of ART, DHA, AS, and DHA7 to AcrB. Surflex-Dock that adopted an empirical scoring function was employed for molecular docking. The crystal structure of AcrB (PDB ID, 2DRD) was obtained from Protein Data Bank. The molecular docking results were analyzed and represented in the PyMOL 1.3 visualization software. Proteins are shown in gray and orange. The compounds are shown as thick sticks colored according to the compound structure (Figure 2A: red, AS; cyan, DHA7; green, ART; yellow, DHA) and the atom type (Figure 2B–E: red, oxygen; cyan, carbon; dark blue, nitrogen), and residues are shown in brown and marked. (**A**) ART and three ART derivatives including DHA, AS, and DHA7 bind to AcrB; (**B**) Longitudinal section of substrate translocation pathway when AS was docked with AcrB; (**C**) Cartoon view of AS docked with AcrB; (**D**) Longitudinal section of substrate translocation pathway when DHA7 was docked with AcrB; (**E**) Cartoon view of DHA7 docked with AcrB.

Previous works verified that AS and DHA7 had antibacterial enhancement activity while ART and DHA did not have such activity [9,10]. Therefore, molecular docking was first performed on ART and three ART derivatives including DHA, AS, and DHA7 to identify a possible binding pocket for their potency. Following this, 86 compounds designed by computer were bound to this pocket to screen the compounds that could bind to this pocket well and might be supposed to have antibacterial enhancement activity. The results showed that AS and DHA7 (with bioactivity) bound at different positions with ART and DHA (without bioactivity) and that the binding position of AS and DHA7 might be important for their bioactivity (Figure 2A).

The binding pocket of AS and DHA7 was lined by hydrophobic (Phe136, Phe178, and Phe615) and polar (Gln176, Ser46, and Tyr327) residues, which were partially overlapped with those of substrates such as minocycline and doxorubicin [11] and inhibitors such as MBX2319 [12] bound to AcrB. AS and DHA7 interacted with a similar site of amino acid residue that was located at the distal binding pocket. The oxygen atom of the peroxide bridge of the nucleus structure and the oxygen atom of the carboxyl group of the side chain of AS interacted with Gln176 and Tyr327 via hydrogen bonding at a distance of 2.1 and 2.0 Å, respectively; and the oxygen atom of carbonyl of the side chain interacted with Phe178 via the electron cloud-stacking effect. The distance between oxygen atom and six carbon atoms on phenyl of Phe178 was 4.4 ± 0.7 Å (Figure 2B,C). The oxygen atom of the six-membered ring of the nucleus structure and nitrogen atom of imidazole of DHA7 interacted with Gln176 and Phe136 via hydrogen bonding at a distance of 2.2 and 3.1 Å, respectively. The oxygen atom of the side chain interacted with Phe178 via the electron cloud-stacking effect. The distance between oxygen atom and six carbon atoms on phenyl of Phe178 was 4.1 ± 0.9 Å (Figure 2D,E). The results demonstrated that Phe136, Gln176, Phe178, and Tyr327 might be the important binding sites in the distal pocket (DP) of AcrB for AS and its analogues which might have antibacterial enhancement activity.

Based on the aforementioned analysis, 86 compounds were docked in the position to which AS and DHA7 bound to get better binding compounds with better antibacterial enhancement activity. The results showed that only 22 compounds bound well to that position (Table S2).

### 2.3. DHA27 from Four Representative Compounds Has the Best Antibacterial Enhancement Activity

**Figure 3:**
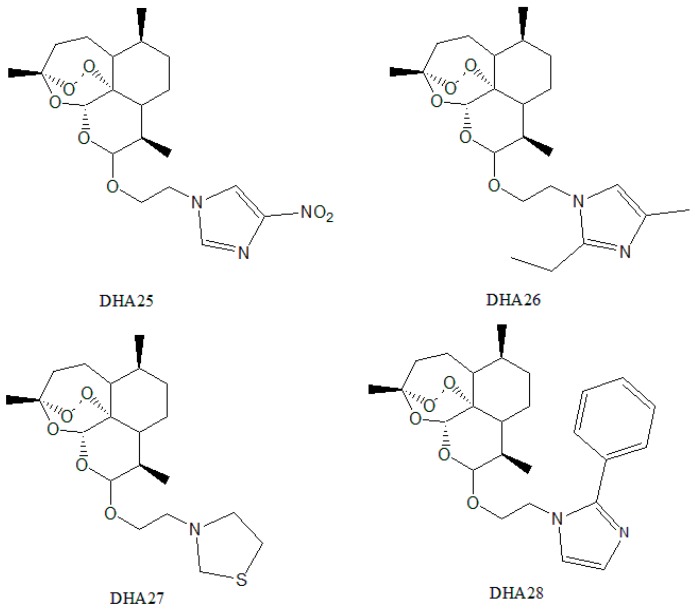
Structure of four representative compounds chosen to be synthesized and named as DHA25–DHA28.

Based on the results of 3D-SAR and molecular docking, four compounds, named as DHA25-DHA28, with a higher total score (Table S3) and simple synthetic routes from 22 compounds were selected and synthesized (Figure 3).

The activities of these compounds were investigated using standard strain *E. coli* ATCC 35218. Because *E. coli* ATCC 35218 is a β-lactamase-producing strain, a β-lactamase inhibitor TZB was added during the MIC test to exclude the disturbing effects of β-lactamase. The results showed that MICs of DHA25-DHA28 alone were higher than 512 mg/L (Table 1A), which meant they had no direct antibacterial activities. When DHA27, DHA7, and AS were combined with AMP, FICI values were 0.31, 0.38, and 0.5, respectively (Table 1B), indicating they all had antibacterial enhancement activity. Among three compounds, DHA27 had the smallest FICI, demonstrating that DHA27 had the best antibacterial enhancement activity.

### 2.4. DHA27 Potentiates Antibacterial Effects of AMP against Not Only E. coli Standard Strain But Also Clinical Strains

DHA27 had the best antibacterial enhancement activity among four compounds, but its aqueous solubility was very poor (maximum solubility was 512 mg/L in 5% DMSO and 128 mg/L in water at room temperature). To carry out subsequent experiments, its aqueous solubility should be improved. The results showed that if DHA27 was converted into corresponding hydrochloride salt, its aqueous solubility markedly improved; the solubility of DHA27 hydrochloride was better than that of DHA27 (<2048 mg/L in water at room temperature).

**Figure 4:**
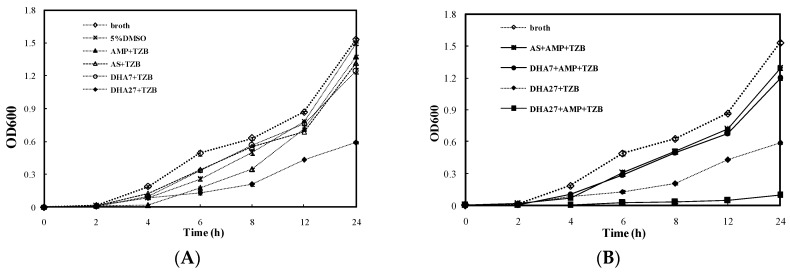
Dynamic growth curves of *E. coli* ATCC 35218. *E. coli* ATCC 35218 from the exponential phase was diluted with LB broth to 1.0 × 10^6^ cfu/mL. Then, 64 mg/L artesunate (AS), DHA7 and DHA27 alone, and in combination with ampicillin (AMP)/tazobactam (TZB) were added to bacterial suspensions. Broth and 5% DMSO were negative control and solvent control, respectively. The bacterial growth was determined by measuring OD_600_ at regular intervals. (**A**) The effect of drugs alone on growth of *E. coli* ATCC 35218; (**B**) The effect of drugs in combination of AMP on growth of *E. coli* ATCC 35218.

For the bacterial dynamic growth curve assay, AS and DHA7 were used as controls. DHA27 hydrochloride (DHA27 for short, 64 mg/L) only slightly delayed the growth of ATCC 35218 (Figure 4A). However, DHA27 in combination with AMP could significantly inhibit the bacterial growth from 4 to 24 h (Figure 4B). This result was also supported by the colony counting method (Figure S3).

Then, 40 *E. coli* clinical strains were used to evaluate the activity of DHA27. The results showed that these clinical strains were highly resistant to AMP and MIC_50_ was 128 mg/L. When 256, 128, and 64 mg/L of DHA27 was used in combination with AMP, the average FICI was 0.36, 0.31 and 0.27, respectively; the effective rate was 47%, 78%, and 66%, respectively (Table 2). The aforementioned results indicated that DHA27 had the antibacterial enhancement activity of AMP against not only *E. coli* standard strain ATCC 35218 but also most clinical strains.

### 2.5. DHA27 Only Potentiates Antibacterial Effects of β-Lactam Antibiotics Not Non-β-Lactam Antibiotics against Clinical E. coli Strains

Previous studies found that AS had a better antibacterial enhancement effect when combined with β-lactam antibiotics than with other kinds of antibiotics. Herein, the antibacterial enhancement activity of DHA27 combined with β-lactam antibiotics and non-β-lactam antibiotics, was also detected. PAβN, as a known AcrB inhibitor, was used as a control the agent.

The results showed that the average FICIs of six strains were almost lower than 0.5 when DHA27 was combined with β-lactam antibiotics including AMP, CPA, OXA, and PIP in a concentration-dependent manner, while FICIs were higher than 0.5 when DHA27 was combined with non-β-lactam antibiotics such as GAT and AZI. Interestingly, PAβN did not work when it was combined with β-lactam antibiotics at every concentration. It only worked when it was combined with GAT and AZI at the highest concentration (128 mg/L); the FICIs were 0.38 and 0.32, respectively (Table 3).

The aforementioned results suggested that DHA27 potentiated the antibacterial effects of β-lactam antibiotics, not non-β-lactam antibiotics, against clinical *E. coli* strains, and DHA27 had better antibacterial enhancement activity when combined with β-lactam antibiotics compared with PAβN.

### 2.6. DHA27 Increases the Accumulation of Daunorubicin and Nile Red within E. coli ATCC 35218 But Does Not Change the Bacterial Membrane Permeability

To investigate the molecular mechanisms of DHA27 was tightly related to AcrB just like AS, two methods were used to observe the effect ofDHA27 on daunorubicin and nile red accumulation.

**Figure 5:**
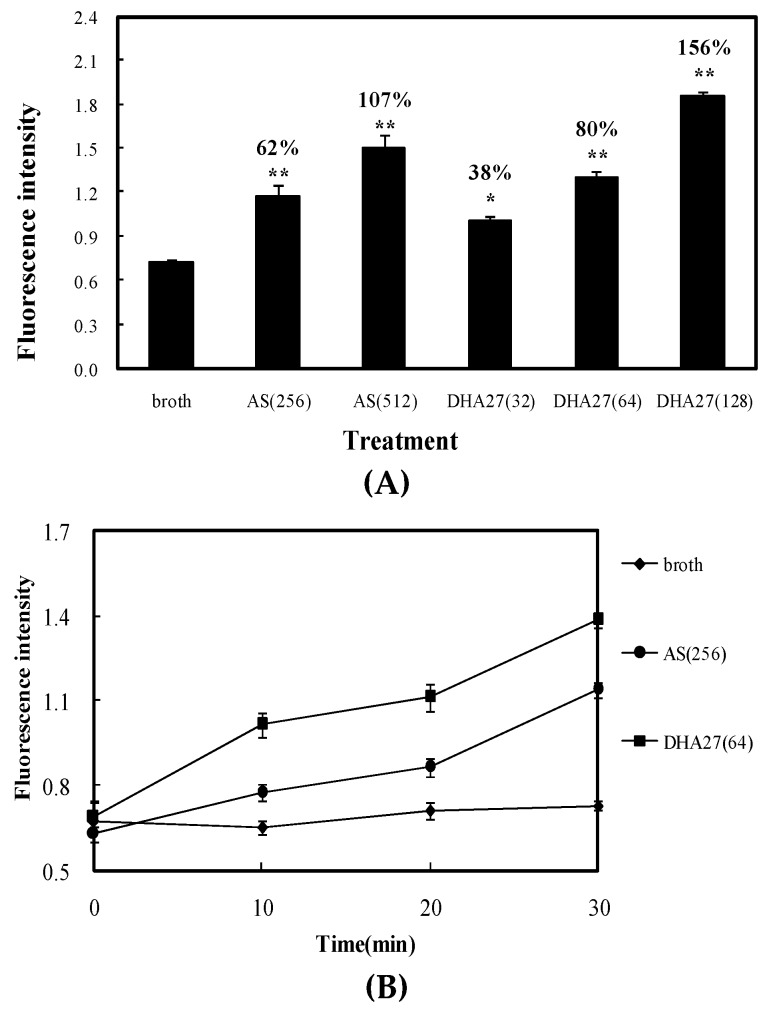
Effect of DHA27 on daunorubicin accumulation within *E. coli* ATCC 35218. Daunorubicin accumulation within *E. coli* ATCC 35218 pretreated with artesunate (AS) and DHA27. (**A**) *E. coli* ATCC 35218 was treated with artesunate (AS 256, 512 mg/L) and DHA27 (32, 64, and 128 mg/L) and cultivated at 37 °C and 100 rpm for 6 h, and then harvested by centrifugation at 4000 rpm for 5 min. After washing three times and resuspending, the bacterial suspension was adjusted to OD_600_ 1.0. Bacteria were incubated with daunorubicin (40 mg/L) in the dark at 37 °C for 30 min. The numerical values on the column were increased percentage of treatment group than broth group; (**B**) *E. coli* ATCC 35218 was treated with artesunate (AS, 256 mg/L) and DHA27 (64 mg/L) and cultivated at 37 °C and 100 rpm for 6 h, and then harvested by centrifugation at 4000 rpm for 5 min. After washing three times and resuspending, the bacterial suspension was adjusted to OD_600_ 1.0. Bacteria were incubated with daunorubicin (40 mg/L) in the dark at 37 °C for 0, 10, 20, and 30 min. Then, 0.5 mL of bacteria was collected. Bacteria were washed three times and resuspended in PBS. Quantitative determination of daunorubicin accumulation in the presence of AS and DHA27 was determined by fluorospectrophotometry at the emission wavelength of 467 nm and excitation wavelength of 588 nm.* *p* < 0.05 compared with broth, ** *p* < 0.01 compared with broth.

Daunorubicin is not a β-lactam antibiotic, and it can emit red autofluorescence [9]; therefore, it can be traced and observed easily and hence used as a tracer agent. The results showed both AS and DHA27 could increase the daunorubicin accumulation within *E. coli* ATCC 35218 in dose- and time-dependent manners, but the effective concentration of DHA27 was much lower than that of AS (Figure 5A,B). Nile red is only a weakly fluorescent in aqueous solution, but undergoes a significant increase in fluorescent quantum yield when in nonpolar environment such as the cell membrane. Cells were preloaded with nile red in the absence or presence of DHA27. Once the cells were energized by the addition of glucose, the efflux of nile red could be observed as a drop in fluorescence. The result was similar to that from daunorubicin accumulation assay. DHA27 could inhibit the efflux of nile red just like AcrB inhibitor PAβN (Figure S5).

As well-known, decreased drug accumulation might be the result of damaged permeability of bacterial membrane. To rule out this possibility of changed permeability of bacterial membrane, the effect of DHA27 on bacterial membrane permeability was observed via examining the hydrolysis rate of a chromogenic β-lactam, nitrocefin by *E. coli* ATCC 35218. Hydrolysis of nitrocefin by the β-lactamase releases a colored compound that can be measured at 490 nm. The rate of nitrocefin hydrolysis is limited by the rate of diffusion across the inner membrane, hence an increased rate of hydrolysis of nitrocefin would be the indicator of decreased membrane permeability. The result showed each concentration of DHA27 (32, 64, and 128 mg/L) did not increase nitrocefin hydrolysis but PAβN (256 mg/L, the effective work concentration) did, demonstrating DHA27 did not change the permeability of bacterial membrane (Figure S6).

Therefore, DHA27 had stronger antibacterial enhancement activity and its bioactivity was closely related to the inhibition of efflux pump and did not affect the bacterial membrane permeability.

### 2.7. DHA27 Loses Antibacterial Enhancement Activity of AMP against E. coli AG100A and Regains it against AG100A/pET28a-AcrB

AcrAB-TolC is one of the most important efflux pumps in *E. coli*. A previous work verified that the antibacterial enhancement activity of AS was associated with the inhibition of AcrB, and DHA27 was also designed targeting AcrB. To verify whether DHA27 was an AcrB inhibitor, *E. coli* AG100A lacking AcrA and AcrB and AG100A/pET28a-AcrB re-expressing AcrB, the recombinant AG100A harboring pET28a-AcrB, were used. PAβN was also used as a control agent.

The results showed that the MICs of AMP, CPA, OXA, PIP, GAT, AZI, PAβN, and DHA27 were high for *E. coli* ATCC 35218 and AG100A/pET28a-AcrB (*E. coli* AG100A transformed with plasmid pET28a-AcrB, Table 4), indicating that the two strains were resistant to these drugs. In contrast, the MICs of all agents except PAβN and DHA27 for *E. coli* AG100A were lower, indicating that AG100A was sensitive and PAβN and DHA27 had no antibiotic effects themselves. When DHA27 was combined with β-lactam antibiotics including AMP, CPA, OXA, and PIP, FICIs were less than 0.5 for ATCC 35218 and AG100A/pET28a-AcrB. When DHA27 was combined with non-β-lactam antibiotics including GAT and AZI, FICIs were more than 0.5 for these two strains. Interestingly, PAβN was just the opposite (Table 4). FICIs were more than 0.5 when DHA27 and PAβN were combined with all antibiotics against *E. coli* AG100A, suggesting that DHA27 and PAβN lost their properties of enhancement of antibacterial effect of antibiotics against strains lacking or with low *acrB* expression but regained their activities against strains with *acrB* expression; DHA27 and PAβN were both AcrB inhibitors.

### 2.8. DHA27Alone Markedly Reduces acrB’s mRNA Expression of E. coli ATCC 35218 in a Dose-Dependent Mannerand Its Antibacterial Enhancement Activity Is Related to the Degree of acrB mRNA Expression in E. coli Clinical Strains

**Figure 6:**
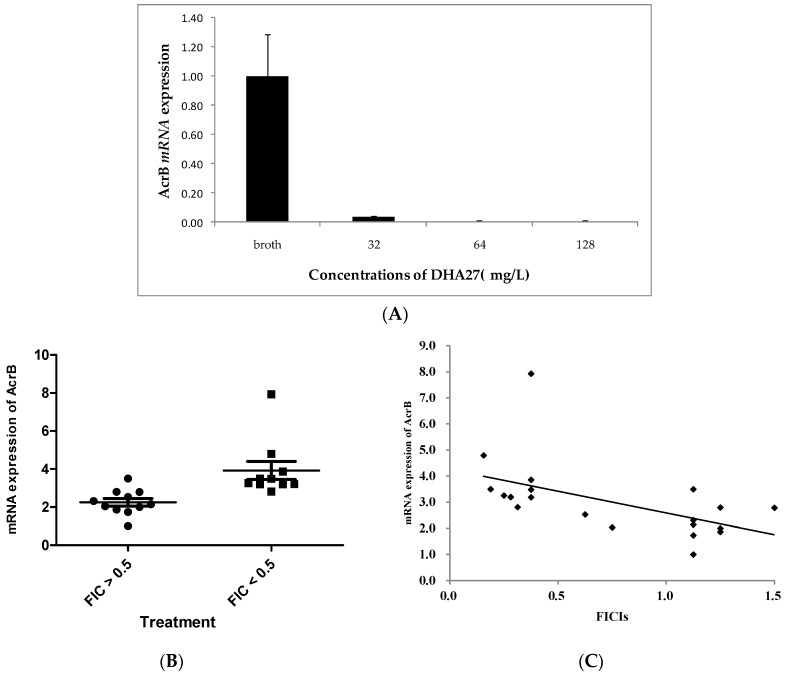
The effect of DHA27 on *acrB* mRNA expression. (**A**) The effect of DHA27 alone on the expression of acrB mRNA expression. *E. coli* clinical strains were treated with different concentration of DHA27 and inoculated into 50 mL of LB broth. Bacteria were cultivated at 37 °C in a heated and shaking environmental chamber of 6 h and then harvested by centrifugation at 1500 rpm for 5 min; (**B**) The *AcrB* mRNA expression of bacteria with FICIs > 0.5 was markedly lower than that of bacteria with FICIs < 0.5; (**C**) *AcrB* mRNA expression has negative correlation with antibacterial enhancement activities of DHA27 against them. *E. coli* clinical strains were inoculated into 50 mL of LB broth. Bacteria were cultivated at 37 °C in a heated and shaking environmental chamber to an OD_600_ of 0.5 and then harvested by centrifugation at 1500 rpm for 5 min. RNA extraction and reverse transcription real-time PCR were performed. The real-time PCR products were detected using a MyiQ Color Fluorescence Real-Time Quantitative PCR kit (Bio-Rad). The gene fold changes were finally calculated according to the 2^−ΔΔ*C*t^ method for each transcript and expressed relative to the values from control group samples.

Previous results showed DHA27 increased daunorubicin and nile red accumulation and lost its antibacterial enhancement activity of AMP in *acrB* knock out strain, and regained antibacterial enhancement activity in *acrB* acquired strain, suggesting the activity of DHA27 was tightly related to AcrB. Therefore, in the present experiment, the effect of DHA27 on the mRNA expression of *acrB* in *E. coli* ATCC 35218 was investigated. The results showed DHA27 alone could markedly down-regulate *acrB* mRNA expression (Figure 6A), suggesting increased daunorubicin accumulation was related to the inhibition of *acrB* mRNA expression.

To further investigate the correlation of DHA27’s activity and *acrB* mRNA expression, the effect of DHA27 on *acrB* mRNA expression was investigated. Ten strains with FICIs lower than 0.5 (DHA27 effective) and another 10 strains with FICIs higher than 0.5 (DHA27 no effective) were chosen, and the *acrB* mRNA expression of these strains was tested. The results showed that *acrB* mRNA expression of these strains with lower FICIs (FICI < 0.5) was higher than that of strains with higher FICIs (FICI > 0.5) (Figure 6B), suggesting that DHA27 had stronger antibacterial enhancement activities against strains with higher *acrB* mRNA expression. Therefore, the aforementioned results further indicated that the degree of *acrB* mRNA expression was related to the bioactivity of DHA27. The correlation coefficient was −0.538 (*p* = 0.012, Figure 6C).

### 2.9. DHA27 Can Bind to Polypeptides from AcrB Obtained by Molecular Docking

Biological experiments demonstrated DHA27 had good antibacterial enhancement activity, which was tightly related to AcrB. Herein, molecular docking was performed to further identify the possible binding sites of DHA27 to AcrB.

**Figure 7:**
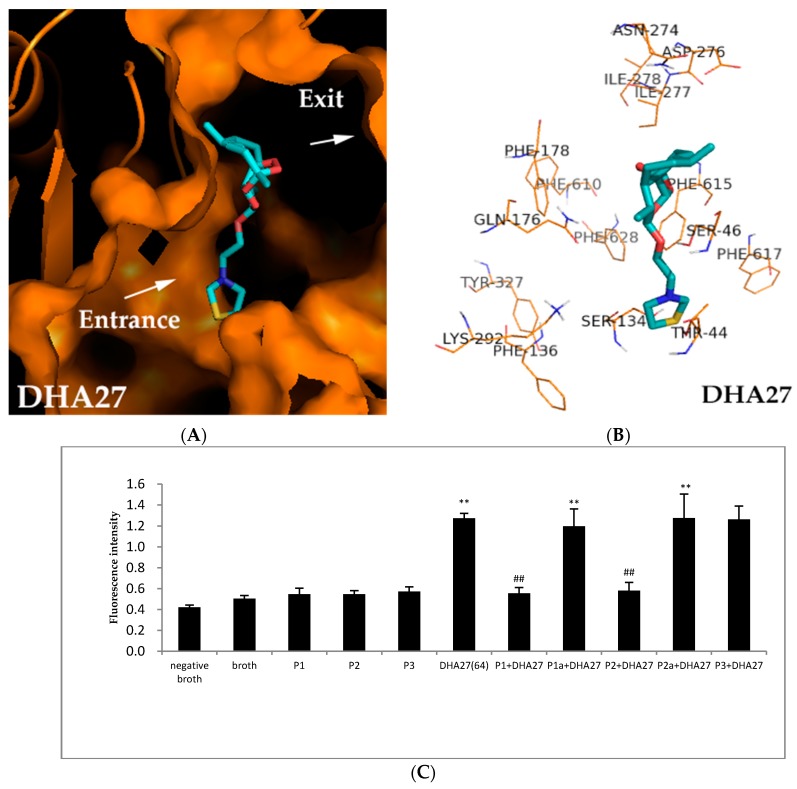
Molecule docking of DHA27 binding to AcrB. The crystal structure of AcrB (PDB ID, 2DRD) was obtained from Protein Data Bank. The molecular docking results were analyzed and represented in the PyMOL 1.3 visualization software. Proteins are shown in orange. The compound is shown as thick sticks colored according to the atom type (red, oxygen; cyan, carbon; white, hydrogen; dark blue, nitrogen, khaki, sulfur), and residues are shown in bluish violet and marked. (**A**) Longitudinal section of substrate translocation pathway when DHA27 was docked with AcrB when PDB ID 2DRD was used; (**B**) Cartoon view of DHA27 docked with AcrB when PDB ID 2DRD was used; (**C**) Effect of DHA27 on binding polypeptides from AcrB and its mutant peptides. Daunorubicin accumulation within *E. coli* ATCC 35218 pretreated with DHA27 and polypeptides; 64 mg/L of DHA27 and polypeptide (molar ratio: 1:1) were cultured for 30 min at 37 °C. This mixture was added to the *E. coli* concentrations of 1.0 × 10^6^ cfu/mL and cultured for 6 h at 37 °C in a heated and shaking environmental chamber. The bacterial pellet was resuspended, and the bacterial suspension was adjusted to an OD_600_ of 1.0. After co-culture with daunorubicin (40 mg/L) in the dark at 37 °C for 30 min, the bacteria were centrifuged at 3000 rpm for 5 min to harvest the bacterial pellet. After washing with PBS (0.2 mM, pH 7.2) five times, fluorospectrophotometry was used to observe daunorubicin accumulation within *E. coli*. The emission wavelength was 467 nm, and the excitation wavelength was 588 nm. ** *p* < 0.01 compared with broth; ^##^ *p* < 0.01 compared with DHA27.

The results showed Gln176, Ser46 and Ser134 were probably involved inthe binding sites of AcrB to DHA27. One of the oxygen atoms of peroxide bridge on the nucleus structure interacted with Gln176. The oxygen atom of the side chain interacted with Ser46 at a distance of 2.1 Å. When PDB ID 2DRD were used, the sulfur atom of the side chain interacted with Ser134 at a distance of 3.1 Å. These interactions were all via the hydrogen binding effect (Figure 7A,B).When PDB ID 28JS and PDB ID 4DX5 were used, similar results were obtained, too (Figure S7). And DHA27 were also surrounded by Phe178, Phe610, Phe615, Phe628 which were in hydrophobic trap (Figure 7B), but there were no formation of chemical bond between DHA27 and these residues. Maybe there was no conjugated structure like benzene ring or carbonyl in DHA27 unlike AS and DHA7; it couldn’t interact with hydrophobic trap through π–π interactions.

To verify the binding sites of DHA27 to AcrB, four polypeptides from AcrB (named as P1, P2, P1a and P2a) were synthesized. P1 and P2 were peptides from the active sites Phe134 and Ser46, respectively. P1a and P2a were respective mutated peptides at Ser134 and Ser46. P3 was an irrelevant peptide from cells. DHA27 was pre-incubated with P1, P2, P1a, P2a and P3, respectively for 30 min, and then pre-incubated DHA27 was added into *E. coli* ATCC 35218. Theoretically, if DHA27 bound the peptides from AcrB, pre-incubated DHA27 would lose its ability to increase the daunorubicin accumulation within ATCC 35218.

The result demonstrated that DHA27 could increase the daunorubicin accumulation within ATCC 35218 as previously mentioned (*p* < 0.01 vs. broth), and P1, P2, and P3 alone couldn’t influence daunorubicin accumulation. DHA27 incubated with P3 didn’t decrease daunorubicin accumulation, demonstrating that DHA27 couldn’t bind to the irrelevant peptide in vitro. However, DHA27 pre-incubated with P1 and P2 lost the activity to increase daunorubicin accumulation, while it pre-incubated with P1a and P2a which were mutant peptides of P1 and P2 regained its activity to increase daunorubicin accumulation, suggesting that DHA27 could bind to P1 and P2 while it could not bind to their mutant peptides, demonstrating Ser134 and Ser46 might be involved in the binding sites of DHA27 (Figure 7C).

### 2.10. DHA27 Has No Cytotoxicity in Cell Proliferation

**Figure 8:**
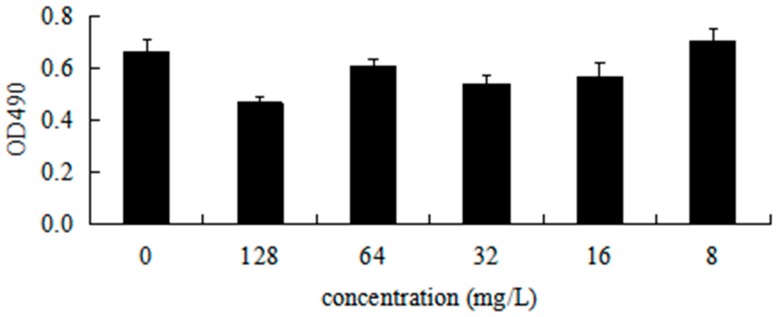
Effect of DHA27 at different concentrations on the growth of mouse macrophages RAW264.7. RAW264.7 cells of logarithmic phase were adjusted to the concentration of 8 × 10^4^ cells per milliliter with the DMEM (10% FBS) culture. The cells were placed on a 96-well plate (180 µL/well) and cultivated at 37 °C, 5% CO_2_ incubator for 24 h. DHA27 (20 µL/well) was added to achieve the final concentrations of 128, 64, 32, 16, and 8 mg/L. The blank culture was set up for control and then the template was put at 37 °C, 5% CO_2_ incubator for 24 h. Mixed 100 μL of low-glucose DMEM and 20 μL of MTS solution were added per well. The plate was placed in an incubator at 37 °C for 1 h at 5% CO_2_. The absorbance was measured at 490 nm.

To investigate the cytotoxicity of DHA27 against mammalian cells, an in vitro cytotoxicity assay was performed using the mouse peritoneal macrophage RAW264.7 cell line. The result showed that 8–64 mg/L of DHA27 could not inhibit the viability of cells up to 24 h, suggesting that DHA27 at concentrations with antibacterial enhancement activity had no cytotoxicity against RAW264.7 cells (Figure 8).

## 3. Discussion

This study was the first to demonstrate that a new derivative from AS, named as DHA27, had good antibacterial enhancement activity against *E. coli* with low cytotoxicity; it was obtained based on the 3D-SAR and molecular docking experiments. It exerted its antibacterial enhancement activity via binding to the DP of AcrB and then inhibiting the function of AcrB.

AcrB plays a key role in the AcrAB-TolC efflux system, inhibition of AcrB can affect the function of AcrAB-TolC, leading to the reverse of bacterial resistance. Till now, the main known AcrB inhibitors are PAβN, NMP, D13-9001, and MBX2319 reported by others, and AS and DHA7 reported by the present study [8,9,10]. However, these inhibitors such as PAβN had much more serious toxicity or lower activities; so new antibacterial enhancers should be developed.

3D-SAR studies have been found to be of great importance to designing and developing potent drugs. CoMFA used for the 3D-SAR methodology is based on the assumption that the changes in antibacterial enhancement activities are related to their 3D shape and changes in molecular properties represented by electrostatic and steric characteristics [13]. The crystal structure of AcrB is clear [11,12,14], it is possible to develop new antibacterial enhancers based on the structure of AcrB. In this study, 86 new compounds having potential antibacterial enhancement activity were designed using 3D-SAR studies. To narrow down the potential active compounds, molecular docking, which could figure out the probable binding pocket of known active compounds, was used. At last, four compounds named as DHA 25-DHA 28 with a higher total score and simple synthetic routes from 22 compounds were selected and synthesized based on the results of 3D-SAR and molecular docking. Among them, DHA 27 had the best antibacterial enhancement activity.

Our previous studies reported the antibacterial enhancement activity of AS and its derivative DHA7 for the first time [9,10]. Although DHA7 had better activity than AS, it was still only effective against standard *E. coli* strains rather than clinical strains and had poor aqueous solubility as AS. Hence, they could not be developed for clinical application. Herein, DHA27 was selected from 86 compounds. Our results demonstrated it could increase the accumulation of daunorubicin and nile red within ATCC 35218 and it didn't increase nitrocefin hydrolysis, demonstrating it played its antibacterial enhancement role via increasing drug accumulation but not changing the bacterial membrane permeability.

It is well known that increased drug accumulation was related to decreased efflux and increased membrane permeability. Nitrocefin is a chromogenic β-lactam which could be hydrolyzed by theβ-lactamase and then released a colored compound that can be measured at 490 nm. The rate of nitrocefin hydrolysis is limited by the rate of diffusion across the inner membrane, hence an increased rate of hydrolysis of nitrocefin would be indicative of inner membrane permeabilization.

*E. coli* AG100A lacking AcrA and AcrB and AG100A/pET28a-AcrB re-expressing AcrB, the recombinant AG100A harboring pET28a-AcrB, were used. The results showed that both DHA27 and PAβN lost the enhancement of AMP against *E. coli* AG100A and regained it against *E. coli* AG100A/pET28a-AcrB, which demonstrated that DHA27 should be an AcrB inhibitor. Moreover, DHA27 alone markedly reduces *acrB’s* mRNA expression of *E. coli* ATCC 35218 in a dose-dependent manner and its antibacterial enhancement activity is related to the degree of *acrB* mRNA expression in *E. coli* clinical strains, which further indicated that DHA27 was an AcrB inhibitor.

DHA27 had the following advantages. (1) The activity exhibited by DHA27 in vitro assays was superior to that of AS and DHA7 at the same concentrations against ATCC 35218; (2) DHA27 was effective against not only *E. coli* standard strains but also clinical strains when it was combined with AMP, which indicated its good potential for clinical application; (3) The activity of DHA27 was much better than that of known AcrB inhibitor PAβN when it was combined with β-lactam antibiotics. β-lactam antibiotics are extensively used worldwide because of their safety. So, DHA27 is more valuable to be developed for clinical use than PAβN; (4) DHA27 could be converted into its hydrochloride so that it had good aqueous solubility for the possibility of its clinical use; (5) DHA27 had lower toxicity than PAβN because it derived from ART [15]. The present data demonstrated that DHA27 had no cytotoxicity at concentrations that had antibacterial enhancement activity. Therefore, above results indicated that DHA27 exhibits the following characteristics of an AcrB inhibitor, as described by Lomovskaya et al. [16]: (i) it inhibits the extrusion or accumulation of AcrAB-TolC substrates; (ii) it potentiates the antibacterial activity of diverse agents that are substrates of AcrAB-TolC; and (iii) it does not exhibit activity against mutants lacking functional AcrAB-TolC pumps.

The crystal structure of AcrB is a homo-trimeric protein, and each protomer is in any of these three conformations: Access (loose or A), Binding (tight or B), and Extrusion (open or C) [11,12]. The substrates are transported through dual multidrug-binding pockets via the peristaltic motion of the substrate translocation pathway [3]. Different substrates bind to different sites of the binding pocket of AcrB. A number of studies [17,18,19] demonstrated that the majority of substrates of AcrB bound to the DP located in the periplasmic domain of the binding conformer [11], suggesting that DP played a major role in the binding and selection of substrates by AcrB [20,21,22]. Some antibiotics have been found to bind to the proximal binding pocket also called the access pocket (AP) in the access protomer [23,24]. AP and DP are separated by a loop rich in glycine called as G-loop.

Several AcrB inhibitors (PAβN, NMP, D13-9001, and MBX2319) and some of its substrates (MIN and DOX) have been found by molecular docking experiments. It has been demonstrated that (1) the binding positions of all inhibitors are partially overlapped [25]. The important binding sites are Phe136, Gln176, Tyr327, Phe178, Phe615, and Phe628, which are also partial sites of binding of AS, DHA7, and DHA27 to AcrB; (2) All inhibitors have higher affinities for the DP of protomer B compared with the substrate MIN. Thus, competitive binding of the inhibitors might alter the properties of substrate-binding sites, reducing the affinity of substrates to the DP of AcrB.

The most likely mechanism of inhibition of DHA27 was through competitive inhibition and/or blockage of access to the substrate-binding site of AcrB. This was in contrast to PAβN, which also increases the permeability of the inner membrane [16]. The binding positions of AS, DHA7, and DHA27 were partially overlapped and a little bit deeper (close to the gate) than those of reported other inhibitors (PAβN, NMP, D13-9001, and MBX2319) [26]. These ART derivatives are apt to interact with polar residues, while other reported inhibitors have a predilection for hydrophobic residues. The reason of this can be the difference in their structure. The structures of ART derivatives have no or only one aromatic nucleus that interacts with polar residues via hydrogen bonding, while the structures of reported inhibitors include more than one aromatic nuclei that can interact with hydrophobic residues via the ring-stacking effect.

The binding sites of DHA27 were partially overlapped with those of AS and DHA7, and previously reported AcrB inhibitors other than MBX2319 and D13-9001 [25]. The binding sites of DHA27 were verified by the competitive inhibition experiments. However, it was not directly confirmed that DHA27 had higher affinity compared with other substrates of AcrB (MIN and DOX). It is proposed that important binding sites for DHA27 were Gln176, Ser46, and Ser134 to influence the morphology of the substrate extrusion channel, alter the properties of substrate-binding sites, and reduce the affinity of substrates to the DP of AcrB. This prediction was supported by the polypeptide-binding assays in vitro.

## 4. Materials and Methods

### 4.1. Reactants and Solvents

Dihydroartemisinine (DHA) was purchased from Holley Wuling Mountain Pharmaceutical Corporation, Ltd. (Chongqing, China). Other synthetic materials were purchased from Aladdin (Shanghai, China). Solvents were purchased from Chuandong Chemical Co., Ltd. (Chongqing, China) and distilled in our laboratory. Daunorubicin hydrochloride for injection was obtained from Pharmacia Italia S.P.A (Milan, Italy).

### 4.2. Bacterial Strains, Antibiotics, Peptides, and Drug Preparation

*E. coli* ATCC 35218 was kept in the laboratory, which was a standard strain resistant to ampicillin (AMP) with high *acrB* mRNA expression. *E. coli* clinical isolates were obtained from the Department of Clinical Laboratory, Southwestern Hospital (Chongqing, China) (sample number 1–40). *E. coli* AG100A lacking AcrAB was donated by Professor Hiroshi Nikaido of the University of California (CA, USA). *E. coli* AG100A/pET28a-AcrB re-expressing AcrB, the recombinant AG100A harboring pET28a-AcrB, was constructed in the laboratory. The information on antibiotics and drugs used in this study is listed in Table S1.

The peptides named as P1 and P2 were obtained from the crystal structure of AcrB (PDB ID, 2DRD). The amino acid sequence of P1 was VSVEKSSSSFLF (number: 126–136), and that of P2 was PVAQYPTIAPPAVTIS (number: 30–46). The peptide of P3 was obtained from other proteins; the amino acid sequence was EECRGRALRLCL. P1a and P2a were mutant peptides mutated at Ser134 and Ser46. The amino acid sequence of P1a was KSSSLTFMVVG (number: 131–140), and that of P2a was PAVTILAAYPG (number: 41–50). All peptides were synthesized by SBS Genetech Co., Ltd. (Beijing, China).

### 4.3. Three-Dimensional Structure–Activity Relationship with Comparative Molecular Field Analysis

Three-dimensional structure–activity relationship (3D-SAR) and the comparative molecular field analysis (CoMFA) were performed using the Sybyl 2.0 molecular modeling software (Tripos International, St. Louis, MO, USA) package, as described previously. In this study, the default CoMFA setting, which included both steric and electrostatic fields, and partial least squares regression analysis were used.

DHA7 was selected as the molecular template, and 18 compounds from previous work were used for database alignment [10]. Molecular energy minimization of each compound was calculated in the compute mode and then stored in the new database. The aligned database molecules, the steric and electrostatic energy values in CoMFA, and the biological activities of these molecules were placed in a spreadsheet. Partial least square (PLS) method was applied to generate 3D-SAR models. The PLS algorithm with the leave-one-out cross-validation method was employed to choose the optimum number of components and assess the statistical significance of each model.

### 4.4. Molecular Docking

Surflex-Dock that adopted an empirical scoring function was employed for molecular docking. The crystal structure of AcrB (PDB ID, 2DRD) was obtained from Protein Data Bank (http://www.rcsb.org). The molecular docking results were analyzed and represented in PyMOL 1.3 visualization software (Program by Warren Lyford DeLano and commercial release by DeLano Scientific LLC).

### 4.5. Synthesis of 12 β-(2-Bromoethoxy) Dihydroartemisinin (Compound **1**)

2-bromoethyl alcohol (1.55 g, 12 mmol) and 50 mL of Et_2_O were added into a 100-mL round-bottomed flask, and then 2 mL of boron trifluoride etherate with ice bath cooling was added. DHA (2.85 g, 10 mmol) was finally added after stirring. The mixture was allowed to react for 5 h and continuously ice bath cooled and stirred. The reaction process was monitored with thin-layer chromatography (TLC). Saturated NaHCO_3_ was added to terminate the reaction. The aqueous layer was extracted with ethyl acetate (EtOAc) (20 mL × 2) after liquid separation, and then the organic layer was merged. The organic layer was washed with 20 mL of saturated saline solution, and then dried with anhydrous Na_2_SO_4_, and the solvent was removed through rotary steaming under a reduced pressure. The raw product was recrystallized with a mixed solvent of petroleum ether and EtOAc, and a 3.122-g white crystal was obtained after filtration and vacuum desiccation. The yield was 79.52% (Figure S1).

### 4.6. Synthesis of DHA25-DHA28

Compound 1 (0.391 g, 1 mmol), K_2_CO_3_ (0.276 g, 2 mmol), and YH (1.2 mmol, Figure S2) were added to a solution of CH_3_CN (30 mL). The mixture was allowed to react at a controlled temperature; the reaction process was monitored by TLC. Then, 15 mL of CH_2_Cl_2_ and 20 mL of saturated NaCl solution were added. The aqueous layer with CH_2_Cl_2_ (10 mL × 2) was extracted after liquid separation, and then the organic layers were merged. The organic layer was washed with 20 mL of saturated saline solution and then dried with anhydrous Na_2_SO_4_; CH_2_Cl_2_ was removed through rotary steaming under a reduced pressure. A pure product was obtained after column chromatography. The yields of DHA25-DHA 28 are shown in Figure S1. The ^1^H-NMR and ^13^C-NMR of DHA25-DHA 28 are shown in Figure S2A.

### 4.7. Synthesis of DHA27 Hydrochloride

DHA27 was dissolved in methanol (50 mL) and converted into the HCl salt by treatment with ethereal HCl. The solvent was evaporated in vacuo. The residue was recrystallized from a mixture of MeOH-hexanes. The ^1^H-NMR of DHA27 hydrochloride is shown in Figure S2B.

### 4.8. Drug Susceptibility Assay

A single colony from Luria-Bertani (LB) agar plates was transferred to sterile liquid LB broth (10 g/L tryptone, 10 g/L NaCl, and 5 g/L yeast extract) and cultivated aerobically in a heated and shaking 50-mL environmental chamber at 37 °C for 12 h. These cultures were then transferred to 500 mL of fresh LB broth for another 12 h. When bacteria were in the exponential phase of growth, they were resuspended and diluted in fresh LB broth to achieve cell growth (1 × 10^6^ cfu/mL) that was subsequently inoculated into 96-well plates. Minimum inhibitory concentrations (MICs) were determined by serial twofold dilutions in LB broth containing different drugs in accordance with the Clinical and Laboratory Standards Institute (CLSI) 2013.

Meanwhile, the MICs of combined drugs were also observed and the fractional inhibitory concentration index (FICI) was calculated according to the method reported previously [26]. FICI < 0.5 = synergy; 0.5–4.0 = irrelevant; > 4.0 = antagonism.

### 4.9. Dynamic Bacterial Growth

Bacteria in the exponential phase of growth were diluted in LB broth to reach a concentration of 1.0 × 10^6^ cfu/mL. According to the MIC results, the same concentrations of AS, DHA7, and DHA27 alone, and in combination with AMP were added to bacterial suspensions. Broth and 5% dimethyl sulfoxide (DMSO) were used without drugs or antibiotic. The bacterial growth was determined by measuring OD_600_ at regular intervals.

### 4.10. Daunorubicin Accumulation within E. coli ATCC 35218

*E. coli* was inoculated into 10 mL of LB broth containing different concentrations of DHA27 and AS. They were cultured for 6 h at 37 °C in a heated and shaking environmental chamber. The bacterial pellet was resuspended, and the bacterial suspension was adjusted to an OD_600_ of 1.0. All the groups were co-cultured with daunorubicin (40 mg/L) in the dark at 37 °C for 0, 10, 20, and 30 min. The bacteria were centrifuged at 3000 rpm for 5 min to harvest the bacterial pellet. After washing with phosphate-buffered saline (PBS) (0.2 mM, pH 7.2) five times, fluorospectrophotometry was used to observe daunorubicin accumulation within *E. coli*. The emission wavelength was 467 nm, and the excitation wavelength was 588 nm.

### 4.11. Nile Red Uptake Assay

*E. coli* 35218 cells were inoculated into 10 mL of LB broth and cultured for 6 h at 37 °C in a heated and shaking environmental chamber. Cells were harvested, and washed in phosphate-buffered saline (PBS) (0.2 mM, pH 7.2) 2 times and resuspended in the same buffer to OD_600_ of 1. CCCP (10 μmol/L) was added to the cells. After culturing for 20 min at 37 °C, DHA27 (128 mg/L) and PAβN (256 mg/L) were added and cultured for another 20 min at 37 °C. Nile red was finally added to a final concentration of 5 μmol/L and cultured for 30 min at 37 °C. The bacteria cells were centrifuged at 3000 rpm for 5 min to harvest the bacterial pellet and resuspended in the PBS, 0.2 mL of this cell suspension was transferred to a plate reader and fluorospectrophotometry was used to observe. The excitation was at 550 nm, emission was at 640 nm. The fluorescence of the cell suspension was followed for 100 s, after which the efflux of nile red was triggered by rapid energization with 50 mmol/L glucose. Fluorescence was monitored for another 200 s.

### 4.12. Nitrocefin Uptake Assay

*E. coli* ATCC 35218 with constitutive expression of chromosomal β-lactamase were grown in LB broth, harvested, and washed in 50 mmol/L potassium phosphate buffer. The cells were subsequently resuspended in the same buffer to OD_600_ of 0.5. The cell suspension was treated with CCCP (10 μmol/L). Different concentrations of DHA27 (32, 64, and 128 mg/L) and PAβN (256 mg/L) were added, respectively. Nitrocefin was then added to give a final concentration of 32 mg/L. Hydrolysis of nitrocefin was monitored by measuring the increase in absorbance at 490 nm with a plate reader.

### 4.13. Competitive Inhibition Assay of DHA27 and Polypeptides from AcrB

DHA27 (64 mg/L) and polypeptide (molar ratio, 1:1) were cultured for 30 min at 37 °C. This mixture was added to the *E. coli* concentrations of 1.0 × 10^6^ cfu/mL, and the next procedure was the same as the daunorubicin accumulation assay.

### 4.14. AcrB mRNA Expression Assay

*E. coli* clinical strains were inoculated into 50 mL of LB broth. Bacteria were cultivated at 37 °C in a heated and shaking environmental chamber to an OD_600_ of 0.5 and then harvested by centrifugation at 1500 rpm for 5 min. RNA extraction and reverse transcription were performed as described previously. The primers were added to the polymerase chain reaction (PCR) tubes and subjected to the real-time PCR. The real-time PCR products were detected using a MyiQ Color Fluorescence real-time Quantitative PCR kit (Bio-Rad, Hercules, CA, USA). The gene fold changes were finally calculated according to the 2^−ΔΔ*C*t^ method for each transcript and expressed relative to the values from control group samples.

### 4.15. MTS Assay

RAW264.7 cells of logarithmic phase were adjusted to the concentration of 8 × 10^4^ cells per milliliter with the Dulbecco’s modified Eagle’s medium (DMEM) (10% fetal bovine serum) culture. The cells were placed on a 96-well plate (180 µL/well) and cultivated in a 37 °C, 5% CO_2_ incubator for 24 h. DHA27 (20 µL/well) was added to achieve the final concentrations of 128, 64, 32, 16, and 8 mg/L. The blank culture was set up for control, and then the template was put in the 37 °C, 5% CO_2_ incubator for 24 h. A mixture of 100 μL of low-glucose DMEM and 20 μL of MTS solution was added per well. The plate was placed in an incubator at 37 °C for 1 h at 5% CO_2_. The absorbance was measured at 490 nm. All data were presented as mean ± standard deviation (*n* = 5).

### 4.16. Statistics and Presentation of Data

Each experiment was repeated at least three times. Each datum point represented the mean of samples, and error bar denoted standard deviations. The Dunnett’s multiple comparison test was used to examine the differences in the dynamic growth of *E. coli* ATCC 35218 and daunorubicin accumulation within *E. coli* ATCC 35218.

SPSS 16.0 (SPSS, Armonk, New York, NY, USA) was used for data analysis. Pearson correlation test was used to examine the relationship of *acrB* mRNA expression in *E. coli* clinical strains and the antibacterial enhancement activities of DHA27 against *E. coli*. A *p* value of 0.05 was considered significant, and a *p* value of 0.01 was considered highly significant.

## 5. Conclusions

In conclusion, DHA27 showed better antibacterial enhancement activity compared with previously reported AcrB inhibitors, and its molecular mechanism might be related to its binding to AcrB in binding conformation. Gln176, Ser46, and Ser134 were probably involved in the binding sites of AcrB. Its favorable physical and pharmacological properties and lack of cytotoxicity at effective concentrations make it a potential efflux pump inhibitor worthy of further investigation.

## Figures and Tables

**Table:** (**A**)

Agents	MIC (mg/L)
AS	>4096
DHA7	1024
DHA-25	>512
DHA-26	>512
DHA-27	1024
DHA-28	>512
AMP + TZB	16

**Table:** (**B**)

Drug Concentrations	FICI
1/8 MIC AS + 1/4MIC AMP	0.50
1/4 MIC DHA7 + 1/4 MIC AMP	0.38
1/2 MIC DHA25 + 1/2 MIC AMP	1.00
1/2MIC DHA26 + 1/4 MIC AMP	0.75
1/16MIC DHA27 + 1/4 MIC AMP	0.31
1 MIC DHA28 + 1/8 MIC AMP	1.13

(**A**) MICs (mg/L) of AS, DHA7, DHA25–DHA28, and AMP alone against *E. coli* ATCC 35218. For this, 96-well plates were incubated for 24 h at 37 °C in an incubator. The MIC values were taken at the lowest drug concentration at which observable growth was inhibited; (**B**) MICs (mg/L) of AS, DHA7, and DHA25–DHA28 in combination with AMP against *E. coli* ATCC 35218. Synergy testing was performed by the checkerboard method. The calculation of FICIs is described in the Materials and method section.

**Table 2:** Effect of DHA27 on antibacterial activity of AMP against 40 clinical *E. coli* strains.

Antibiotic	*n*	MIC_50_	Concentration of DHA27 (mg/L)					
			256		128		64	
			FICI	Effective Rate	FICI	Effective Rate	FICI	Effective Rate
AMP + TZB	40	128	0.36	47%	0.31	78%	0.27	65%

Synergy testing was performed for 40 clinical *E. coli* strains by the checkerboard method. Studies of synergistic activities of different concentrations of DHA27 and ampicillin (AMP)/tazobactam (TZB) were performed. The calculation of FICI and the explanation are described in the Materials and methods section. FICI, Fractional inhibitory concentration index. Effective rate = the number of strains which DHA27 or PAβN has antibacterial enhancement activity/Total number of tested strains.

**Table 3:** Effect of DHA27 and PAβN on antibacterial effects of six antibiotics on six *E. coli* strains.

Strains	n	Antibiotics	MIC_50_	Concentration of DHA27/PAβN (mg/L)											
				32				64				128			
				DHA27		PAβN		DHA27		PAβN		DHA27		PAβN	
				FICI	Effective Rate	FICI	Effective Rate	FICI	Effective Rate	FICI	Effective Rate	FICI	Effective Rate	FICI	Effective Rate
*E. coli* Clinical strains	6	AMP + TZB	32	0.48	34%	0.67	0%	0.21	100%	0.64	0%	0.24	100%	0.61	33%
		CPA + TZB	256	0.45	50%	0.69	0%	0.33	83%	0.72	0%	0.23	100%	0.56	0%
		OXA + TZB	256	0.42	50%	0.78	0%	0.32	83%	0.74	0%	0.29	83%	0.51	33%
		PIP + TZB	8	0.53	50%	0.82	0%	0.24	100%	0.69	0%	0.26	100%	0.84	0%
		GAT	2	0.76	0%	0.65	17%	0.59	0%	0.51	33%	0.58	17%	0.38	83%
		AZI	32	0.83	0%	0.93	0%	0.68	17%	0.42	50%	0.52	50%	0.32	83%

Synergy testing was performed for six *E. coli* strains by the checkerboard method. Studies of synergistic activities of different concentrations of dihydroartemisinine 27 (DHA27), phe-Arg β-naphthylamide (PAβN), and several kinds of antibiotics, including ampicillin (AMP), cefpiramide (CPA), oxacillin (OXA), piperacillin (PIP), gatifloxacin (GAT), and azithromycin (AZI), were performed. The calculation of FICI and the explanation are described in the Materials and methods section. FICI, Fractional inhibitory concentration index. Effective rate = The number of strains which DHA27 and PAβN has antibacterial enhancement activity against them/Total number of tested strains.

**Table 4:** Effect of DHA27 and PAβN in combination with different antibiotics on antibacterial activity against three *E. coli* strains.

Antibiotics	*E. coli* ATCC 35218 *E. coli* AG100 *E. coli* AG100A/pET28a-AcrB								
		64 mg/L			64mg/L			64 mg/L	
	No Drug	DHA27 ^a^	PaβN ^b^	No Drug	DHA27 ^c^	PaβN ^d^	No Drug	DHA27 ^e^	PaβN ^f^
	MIC	FICI	FICI	MIC	FICI	FICI	MIC	FICI	FICI
AMP	32	0.31	0.75	2	1.06	1.13	512	0.31	0.75
CPA	8	0.19	1.25	0.5	0.56	1.13	4	0.31	1.25
OXA	128	0.13	1.25	8	0.56	1.13	512	0.09	0.5
PIP	8	0.19	0.75	0.25	1.06	1.13	4	0.38	1.25
GAT	0.0625	1.06	0.38	0.016	1.06	1.13	0.0625	1.06	0.5
AZI	32	0.56	0.28	0.5	1.06	0.63	32	0.56	0.38

^a^ The MIC of DHA27 against *E. coli* ATCC 35218 is 1024 mg/L; ^b^ The MIC of PaβN against *E. coli* ATCC 35218 was 256mg/L; ^c^ The MIC of DHA27 against *E. coli* AG100 was 1024 mg/L; ^d^ The MIC of PAβN against *E. coli* AG100 was 512 mg/L; ^e^ The MIC of DHA27 against *E. coli* AG100A/pET28a-AcrB was 1024 mg/L; ^f^ The MIC of PAβN against *E. coli* AG100A/pET28a-AcrB was 256 mg/L. Synergistic activities of DHA27 and PAβN in combination with antibiotics on three *E. coli* strains were tested by checkerboard method. The calculation of FICIs was described in the Materials and method section. FICI, Fractional inhibitory concentration index; MIC, minimum inhibitory concentration.

## Supplementary Materials

Supplementary materials can be found at www.mdpi.com/1422-0067/17/11/1934/s1.
