# Supplementary material for: Design of New Antibacterial Enhancers Based on AcrB’s Structure and the Evaluation of Their Antibacterial Enhancement Activity
Source: Int J Mol Sci. 2016 Nov 18;17(11):1934. doi: 10.3390/ijms17111934 (PMC5133929; doi:10.3390/ijms17111934)
Supplement: Supplementary file 1 [file ijms-17-01934-s001.pdf]

upplementary Materials: Design of New Antibacterial Enhancers Based on AcrB's Structure and the Evaluation of Their Antibacterial Enhancement Activity

Yi Song, Rongxin Qin, Xichun Pan, Qin Ouyang, Tianyu Liu, Zhaoxia Zhai, Yingchun Chen, Bin Li and Hong Zhou

Table S1. The statistical parameters of CoMFA.

PLS Statistics	Calibration Set
R squared [a]	0.993
Q squared [b]	0.589
N [c]	7
Standard error of estimate [d]	0.065
F value [e]	214.234

Statistical parameters of partial least square (PLS) analysis. The PLS method was applied to generate 3D-SAR models. The PLS algorithm with the leave-one-out cross-validation method was employed to choose the optimum number of components and assess the statistical significance of each model; [a] Correlation coefficient squared of PLS statistics. The correlation coefficient between the calculated and experimental activities of non-cross validated value (r^2) of 0.993 indicates that the fitness of analyzed results is 99% compared to experimental results, which is better when itisapproachingto1; [b] Leave-one out cross-validated PLS analysis. Q squared (q^2) can evaluated the predictive ability of CoMFA models; models are considered to be good predictive power when q^2 is >0.5; [c] Optimum number of components obtained from cross-validated PLS analysis, which is better when it is >5; [d] It is better when it is lower; [e] *F* value indicates the significant difference between the effective samples, which are better when it was between 100 and 600.

Table S2. The information on drugs and antibiotics were used in this study.

Drugs and Antibiotics	Abbreviation	Company	Company Location
Ampicillin	AMP	Genview	Beijing, China
Cefpiramide	CPA	Guangzhou Baiyun Mountain Pharmaceutical Limited by Share Ltd.	Guangzhou, China
Oxacillin	OXA	Southwest Pharmaceutical Limited by Share Ltd.	Chongqing, China
Piperacillin	PIP	Runze Pharmaceutical Co., Ltd.	Suzhou, China
Gatifloxacin	GAT	Luo Xin Pharmaceutical Group Co., Ltd.	Shandong, China
Azithromycin	AZI	Pfizer Pharmaceuticals Ltd.	NY, USA
Tazobactam	TZB	Sigma	MO, USA
Phe-Arg β-naphthylamide	PAβN	Sigma	MO, USA
Injectable artesunate	AS	Guilin No. 2 Factory	Guangxi, China
Dihydroartemisinin 7	DHA7	Synthesized by the present group	Chongqing, China

Table S3. Surex dock scores (kcal/mol) of 22 derivatives.

Comp.	Total_Score [a]	Crash [b]	Polar [c]	D_Score [d]	PMF_Score [e]	G_Score [f]	Chem-Score [g]
AS	6.2796	−0.5519	1.2656	−126.4052	27.1845	−211.0594	−13.1069
DHA7	6.4801	−0.7569	1.1956	−113.6589	26.6443	−213.1945	−12.3003
1	5.5392	−0.7392	1.0363	−128.8579	48.8487	−227.5580	−11.2945
2	6.5472	−2.1289	1.0052	−133.8129	53.6052	−264.8193	−18.5962
3	5.6351	−0.8576	0.9578	−136.0724	39.6413	−251.7083	−11.8375
4	7.1389	−1.2385	1.1308	−125.4045	27.2729	−252.9736	−13.8301
**5**	7.4427	−0.9113	1.1125	−132.4229	50.5110	−236.8362	−15.5238
6	5.6872	−2.5676	3.3082	−143.0767	60.6718	−260.4081	−14.0520
7	8.2818	−2.1528	1.0461	−157.3601	63.4364	−298.3658	−21.1956
8	6.5239	−0.9137	3.0655	−100.9698	32.4657	−159.0269	−11.3432
9	4.8809	−1.7189	2.2861	−129.3709	12.7769	−203.3223	−11.7440
10	6.6730	−1.1369	2.3830	−120.7153	4.0765	−229.6481	−11.2000
**11**	6.8374	−0.9227	1.0169	−129.6429	70.2738	−216.2878	−10.5319
12	6.9131	−1.4056	2.0906	−120.1748	60.5831	−201.3377	−12.3554
**13**	7.0232	−0.6907	2.0110	−111.6907	38.6349	−176.7910	−9.0069
14	6.9304	−1.3128	3.0584	−121.9317	48.9783	−240.1601	−14.0234
15	7.0360	−2.3959	0.3376	−149.0298	49.3567	−279.2221	−14.3946
16	7.1538	−0.9684	1.2451	−123.1439	27.9912	−215.4057	−10.5446
17	7.3183	−0.7987	1.0366	−105.3284	31.8395	−186.6331	−9.2670
18	7.3700	−1.6393	2.9500	−123.0859	49.1846	−221.6328	−7.1051
19	7.4870	−1.8642	1.6572	−154.0849	50.9602	−240.9163	−7.1970
**20**	8.2178	−0.9082	1.1268	−155.4337	34.8473	−281.2133	−16.7161
21	6.4989	−1.0150	2.3142	−133.1558	41.7465	−213.2861	−21.8276
22	5.2221	−2.6124	2.2555	−145.5035	73.2220	−272.4852	−6.4815

Compounds (abbreviated as Comp.) with underlines were chosen to be synthesized; [a] Total Score (Consensus Score) integrates a number of popular scoring functions for ranking the affinity of ligands bound to the active site of a receptor; [b] Crash score revealing the inappropriate penetration into the binding site. Crash scores close to 0 are favorable. Negative numbers indicate penetration; [c] Polar indicating the contribution of polar interactions to the total score; [d] D-score for charge and van der Waals interactions between the protein and the ligand; [e] PMF-score indicating Helmholtz free energies of interactions for protein-ligand atom pairs (potential of mean force, PMF); [f] G-score showing hydrogen bonding, complex (ligand-protein), and internal (ligand-ligand) energies; [g] Chem-score points for H-bonding, lipophilic contact, and rotational entropy, along with an intercept term.

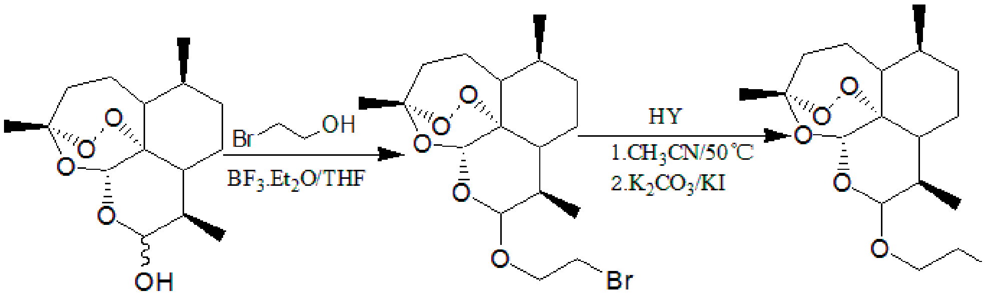

compound1

Agents	YH	M1/mmol	YH/mmol	K_2CO_3/mmol	Temperature/°C	Time/h	Yield/%
**DHA25**	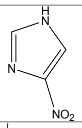	1	1.2	2	0.1	50	64.05
**DHA26**	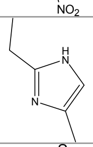	1	1.2	2	0.1	50	40.28
**DHA27**	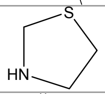	1	1.2	2	0.1	50	32.65
**DHA28**	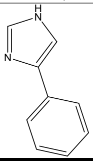	1	1.2	2	0.1	50	48.88

Figure S1. Synthetic routes and yields of DHA25-DHA28.

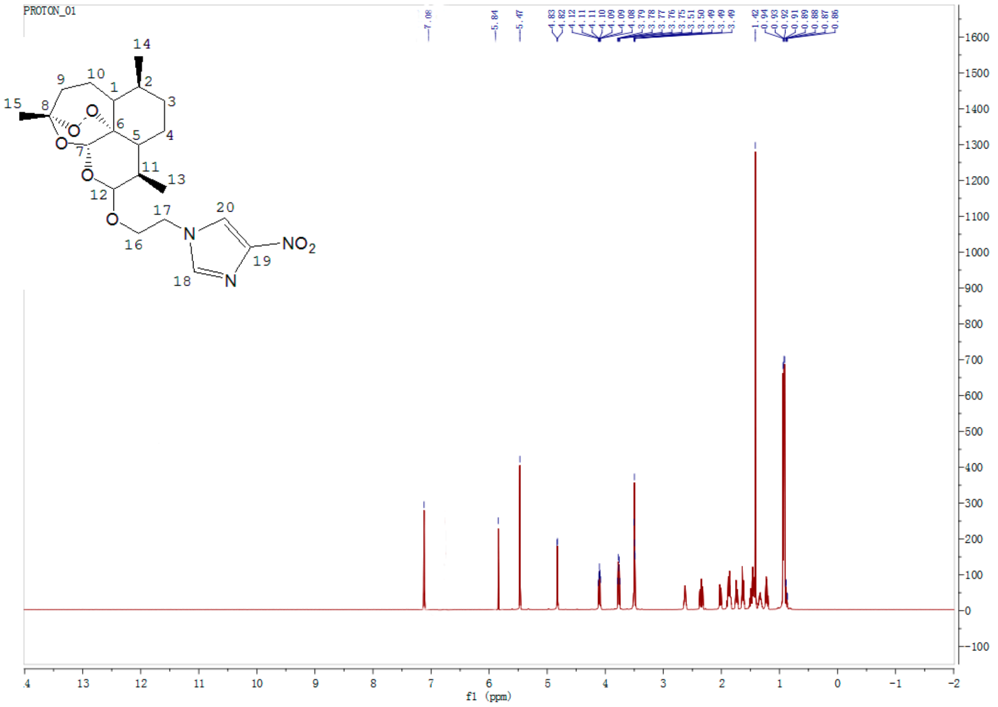

12 β-(2-(4-Methyl-1H-imidazol-1-yl) ethoxy) dihydroartemisinin (DHA25): Yield: 64.05%; Yellow oily; ^{1}H-NMR (600 MHz, $CDCl_3$) δ ppm: 0.91–0.94 (6 H, d, H-13, H-14), 1.42 (3 H, s, H-15), 1.24–2.04 (10 H, m, H-2, H-3, H-4, H-7, H-9 and H-10), 2.35 (1H, m, H-1), 2.61 (1 H, m, H-11), 3.48–3.5 (1 H, m, H-16), 3.75–3.77 (1 H, m, H-12), 4.08–4.11 (2 H, m, H-16 and H-17), 4.82 (1 H, s, H-17), 5.47 (1 H, s, H-5), 5.84 (1 H, s, H-20), 7.08 (1 H, s, H-18); HRMS calculated for $C_{20}H_{29}N_3O_7$ (M + H) + 423.2228, found 423.2221.

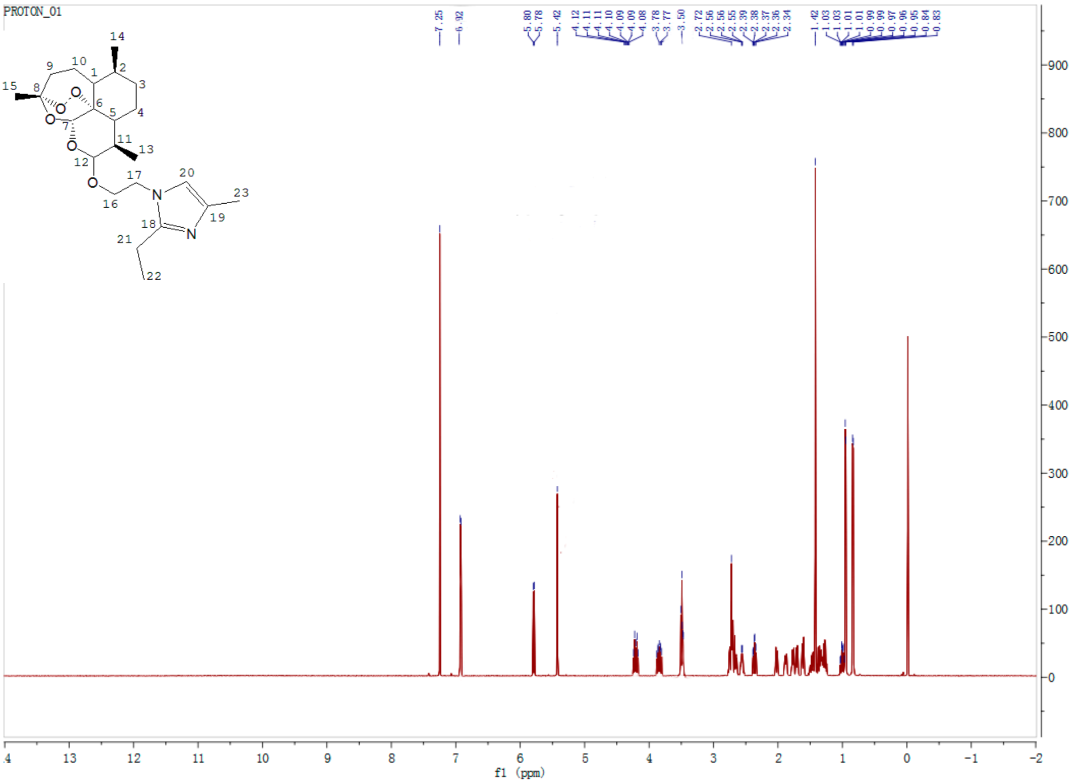

12 β-(2-(2-ethyl-4-Methyl-1H-imidazol-1-yl) ethoxy) dihydroartemisinin (DHA26): Yield: 40.28%; Yellow oily; ^{1}H-NMR (600 MHz, $CDCl_3$) δ ppm: 0.83 (3 H, d, H-14), 0.95 (3 H, d, H-13), 1.42 (3 H, s, H-15), 1.24–2.04 (10 H, m, H-2, H-3, H-4, H-7, H-9 and H-10), 2.72 (3 H, s, H-23), 2.34–2.36 (1 H, m, H-1), 2.56 (1 H, m, H-11), 3.50 (1 H, m, H-16), 3.77–3.78 (1 H, m, H-12), 4.08–4.12 (2 H, m, H-16 and H-17), 4.82 (1 H, s, H-17), 5.42 (1 H, s, H-5), 5.80 (1 H, s, H-20), 6.92 (2 H, s, H-21), 7.25 (3 H, d, H-22); HRMS calculated for $C_{23}H_{36}N_2O_5$ (M + H) + 421.2384, found 421.2386.

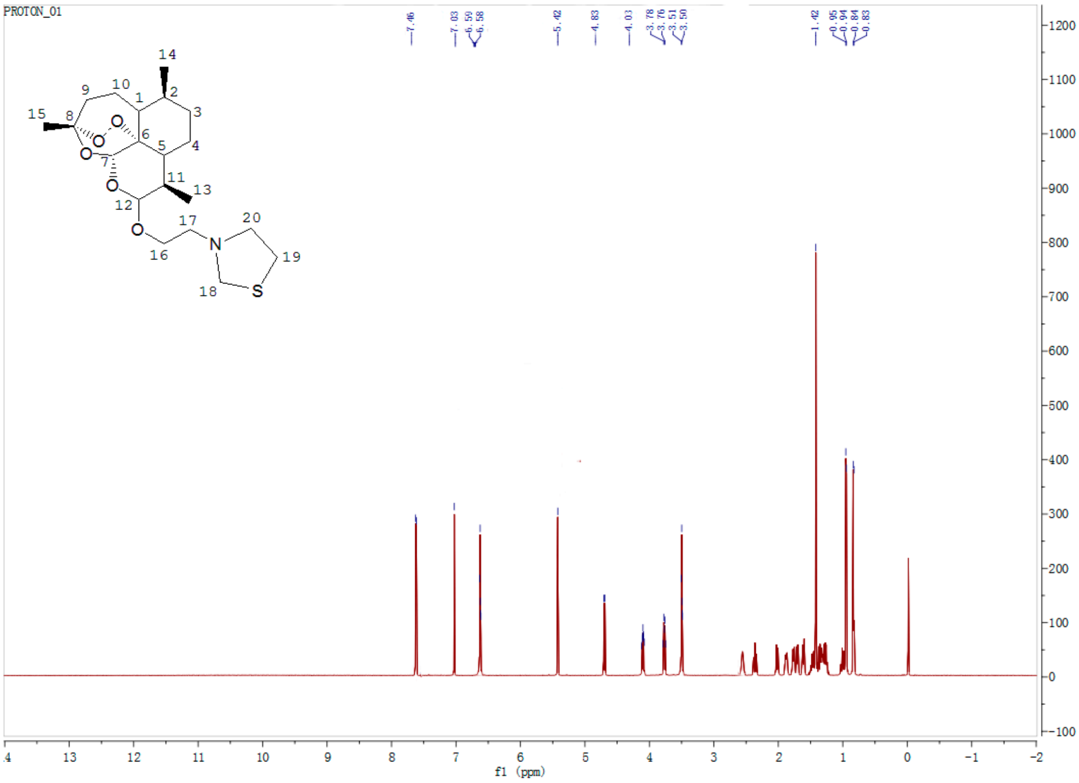

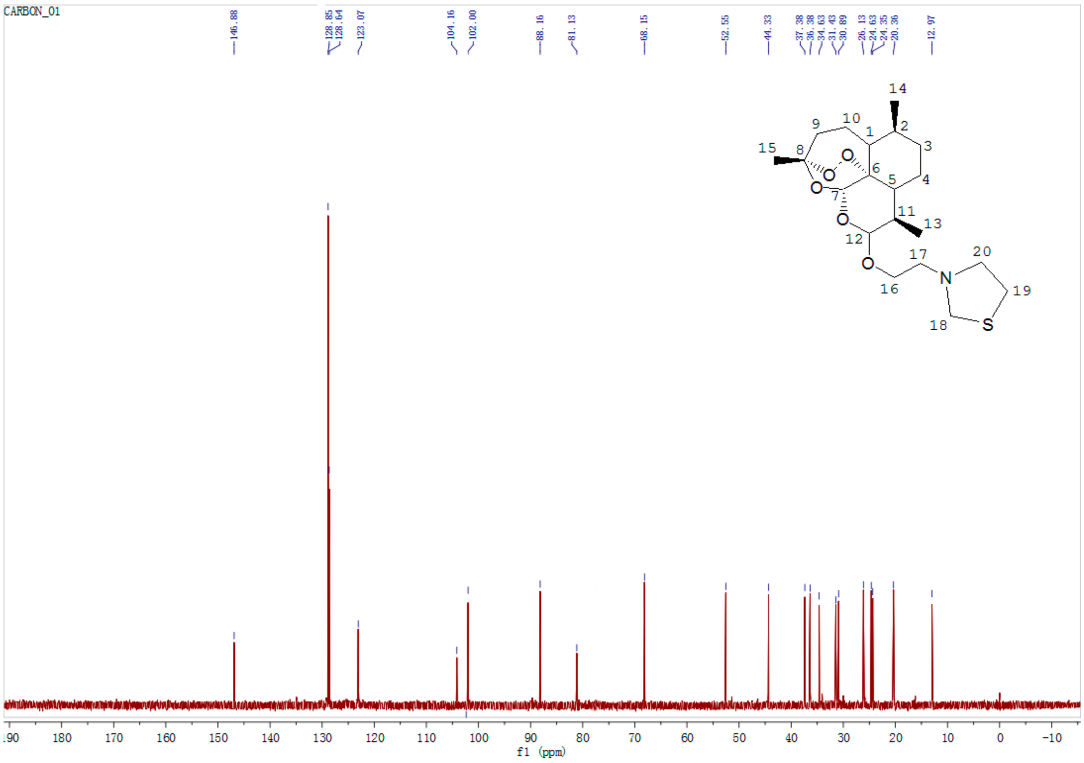

12 β-(2-(1H-thiazole-1-yl) ethoxy) dihydroartemisinin (DHA27):Yield: 32.65%; yellow oil; ^{1}H-NMR (600 MHz, $CDCl_3$) δ ppm: 0.83–0.95 (6 H, d, H-13, H-14), 1.42 (3 H, s, H-15), 1.23–2.04 (10 H, m, H-2, H-3, H-4, H-7, H-9 and H-10), 2.35 (1 H, m, H-1), 2.61 (1 H, m, H-11), 3.50-3.51 (1 H, m, H-16), 3.76–3.78 (1 H, m, H-12), 4.03 (3 H, m, H-16 and H-17), 4.83 (1 H, s, H-12), 5.42 (1 H, s, H-5), 6.58–6.59 (2 H, s, H-19), 7.03 (2 H, s, H-18), 7.46 (2 H, s, H-20); ^{13}C-NMR (150 MHz, $CDCl_3$) δ ppm: 128.9 (C-20), 128.6 (C-19), 123.1 (C-18), 104.2 (C-4), 102.0 (C-12), 88.2 (C-5), 81.2 (C-6), 68.2 (C-16), 52.6 (C-1), 44.3 (C-7), 37.4 (C-11), 36.4 (C-10), 34.7 (C-3), 31.4 (C-17), 30.9 (C-9), 26.1 (C-8), 24.6 (C-15), 24.4 (C-2), 20.4 (C-14), 13.0 (C-13); HRMS calculated for $C_{20}H_{33}NO_5S{\cdot}HCl$ (M + H) + 399.5721, found 399.5726.

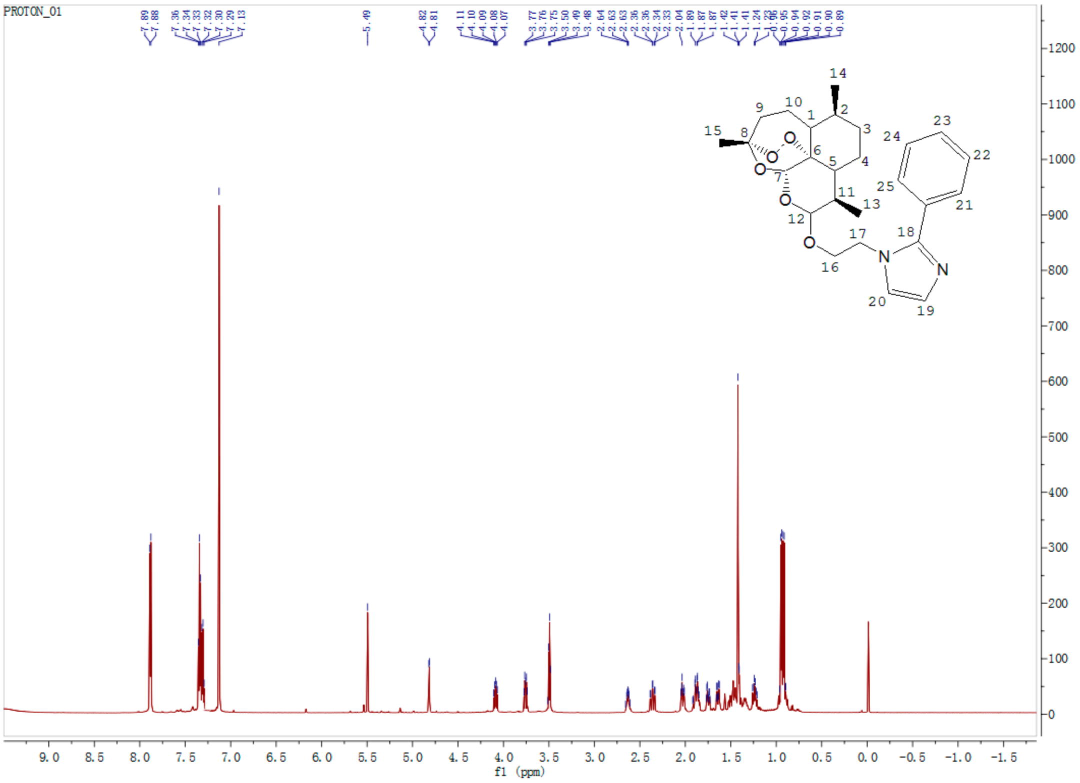

(**A**)

12 β-(2-(2-Phenyl-1H-imidazol-1-yl) ethoxy) dihydroartemisinin (DHA28): Yield: 48.88%; Yellow oily; ^{1}H-NMR (600 MHz, $CDCl_3$) δ ppm: 0.89–0.96 (6 H, d, H-13, H-14), 1.41 (3 H, s, H-15), 1.24–2.04 (10 H, m, H-2, H-3, H-4, H-7, H-9 and H-10), 2.33–2.36 (1 H, m, H-1), 2.63 (1 H, m, H-11), 3.48–3.5 (1 H, m, H-16), 3.75–3.77 (1 H, m, H-12), 4.07–4.11 (2 H, m, H-16 and H-17), 4.81 (1 H, s, H-17), 5.49 (1 H, s, H-5), 7.13 (2 H, s, H-19, H-20), 7.29–7.89 (5 H, m, Ar); HRMS calculated for $C_{25}H_{34}N_2O_5$ (M + H) + 454.5832, found 454.5835.

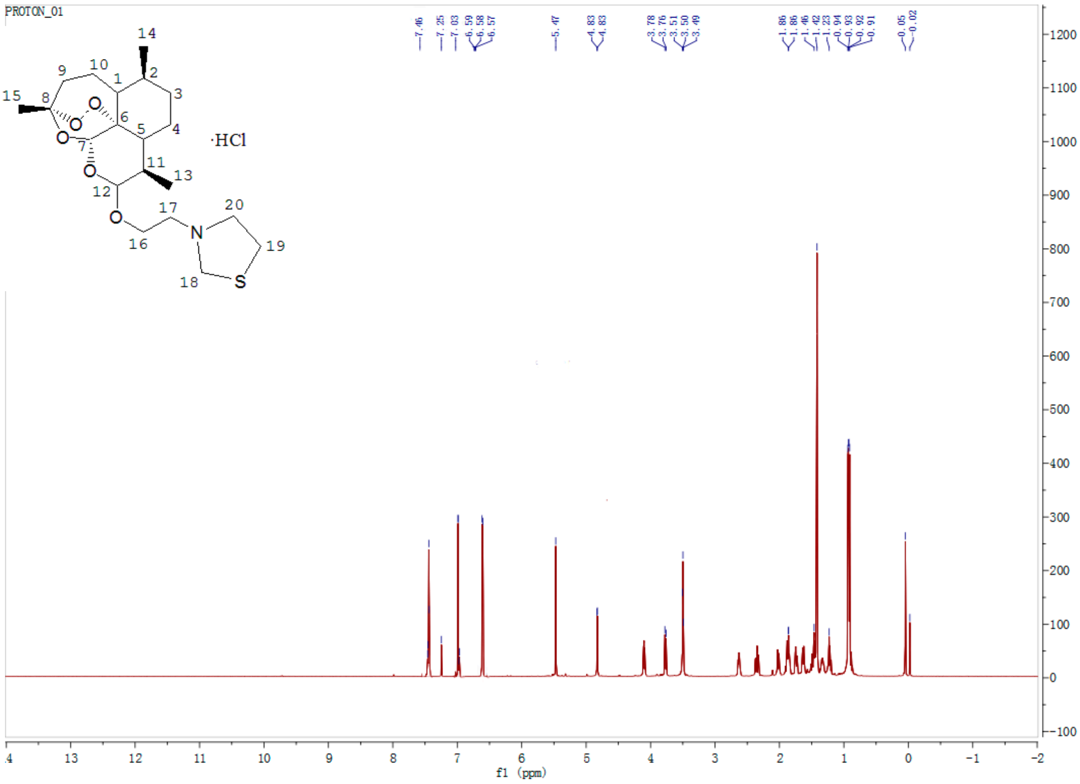

(**B**)

12 β-(2-(1H-thiazole-1 yl) ethoxy) dihydroartemisinin monohydrochloride (DHA27 hydrochloride): Yield: 85.9%; white crystal; ^{1}H-NMR (600 MHz, $CDCl_3$) δ ppm: 0.91–0.94 (6 H, d, H-13, H-14), 1.42 (3 H, s, H-15), 1.23–2.04 (10 H, m, H-2, H-3, H-4, H-7, H-9 and H-10), 2.35 (1 H, m, H-1), 2.61 (1 H, m, H-11), 3.49-3.51 (1 H, m, H-16), 3.76–3.78 (1 H, m, H-12), 4.08–4.11 (3 H, m, H-16 and H-17), 4.83 (1 H, s, H-12), 5.47 (1 H, s, H-5), 6.57–6.59 (2 H, s, H-19), 7.03 (2 H, s, H-18), 7.46 (2 H, s, H-20). HRMS calculated for $C_{20}H_{33}NO_5S{\cdot}HCl$ (M + H) + 436.0784, found 436.0776.

Figure S2. ^{1}H-NMR and ^{13}C-NMR of DHA25–28 and DHA27 hydrochloride. (**A**) ^{1}H-NMR and ^{13}C-NMR of DHA25-28 (**B**)^{1}H-NMR of DHA27 hydrochloride.

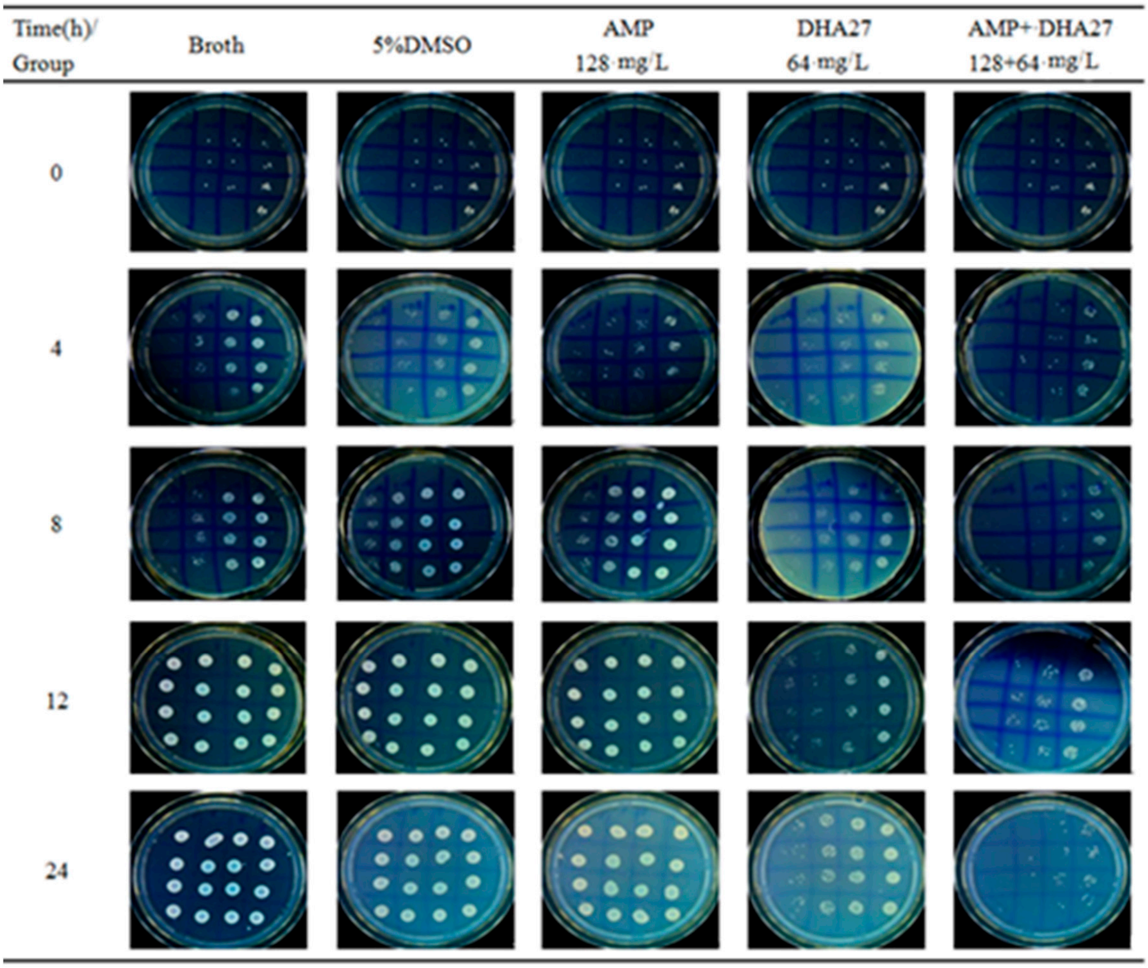

Figure S3. Effect of DHA27 alone and in combination with AMP against *E. coli* ATCC 35218. *E. coli* ATCC35218 was inoculated into 10 mL of LB broth and was divided into five groups containing broth, 5% DMSO, ampicillin (AMP) alone, DHA27 alone, and DHA27 in combination with ampicillin. Bacteria were cultivated at 37 °C in a heated and shaking environmental chamber for 24 h. Bacterial liquid was smeared in the culture dishes at regular time points at 0, 4, 8, 12, and 24 h. Culture dishes were cultivated at 37 °C in a heated environmental chamber, and bacterial colony growth was observed.

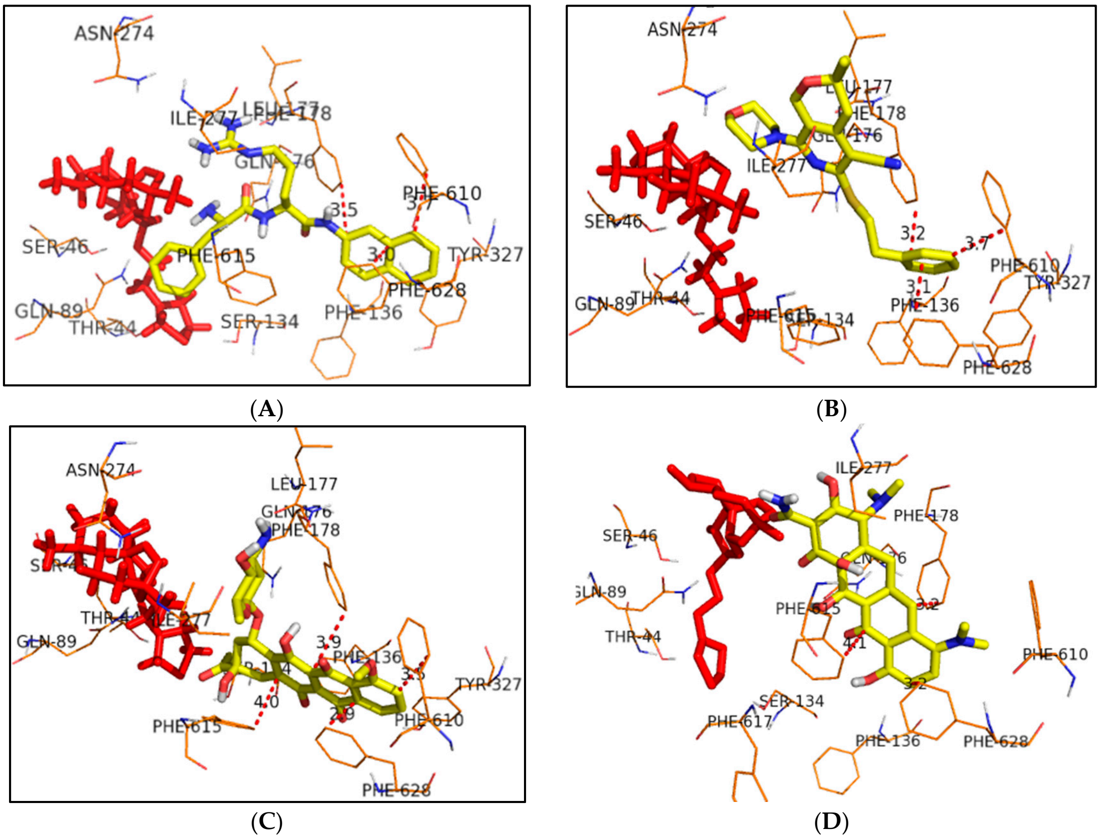

Figure S4. Comparison of the binding sites of DHA27, PAβN, MBX2319, DOX and MIN in AcrB. Surflex-Dock that adopted an empirical scoring function was employed for molecular docking. The crystal structure of AcrB (PDB ID, 2DRD) was obtained from Protein Data Bank. The molecular docking results were analyzed and represented in the PyMOL 1.3 visualization software. Proteins are shown in green. The compounds are shown as thick sticks colored according to the compound structure and the atom type. (**A**) Cartoon view of DHA27 and PAβN docked with AcrB; (**B**) Cartoon view of DHA27 and MBX2319 docked with AcrB; (**C**) Cartoon view of DHA27 and DOX docked with AcrB; (**D**) Cartoon view of DHA27 and MIN docked with AcrB. DHA27 are red in all pictures.

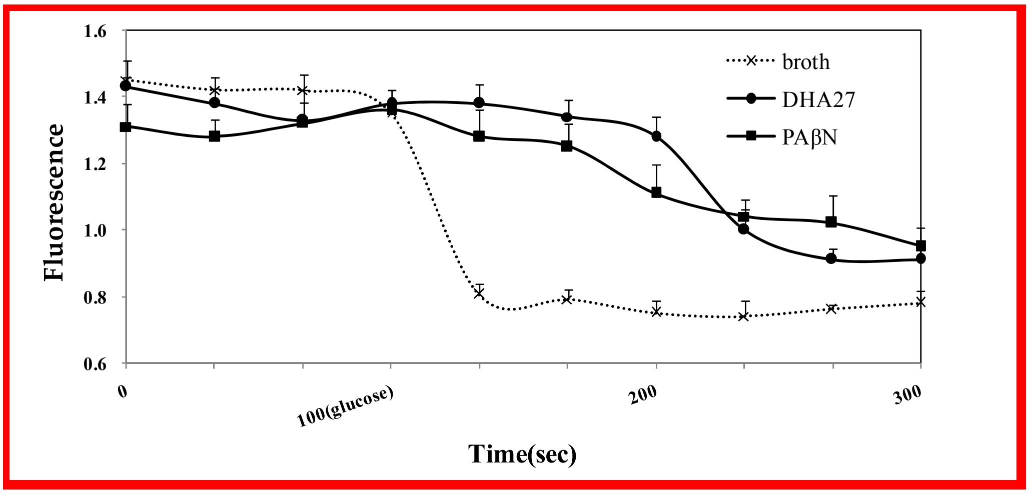

Figure S5. Effect of DHA27 on nile red accumulation within *E. coli* ATCC 35218. *E. coli* ATCC35218 were inoculated into 10 mL of LB broth and cultured for 6 h at 37 °C in a heated and shaking environmental chamber. Cells were harvested, and washed in phosphate-buffered saline (PBS) (0.2 mM, pH 7.2) 2 times and resuspended in the same buffer to OD_{600} of 1. CCCP (10 μmol/L) was added to the cells. After cultured for 20 min at 37 °C, DHA27 (128 mg/L) and PaβN (256 mg/L) were added and cultured for another 20 min at 37 °C. Nile Red was finally added to a final concentration of 5 μmol/L and cultured for 30min at 37 °C. The bacteria cells were centrifuged at 3000 rpm for 5 min to harvest the bacterial pellet and resuspended in the PBS, 0.2 mL of this cell suspension was transferred to a plate reader and fluorospectrophotometry was used to observe. The excitation was at 550 nm, emission was at 640 nm. The fluorescence of the cell suspension was followed for 100 s, after which Nile Red efflux was triggered by rapid energization with 50 mmol/L glucose. Fluorescence was monitored for another 200 s.

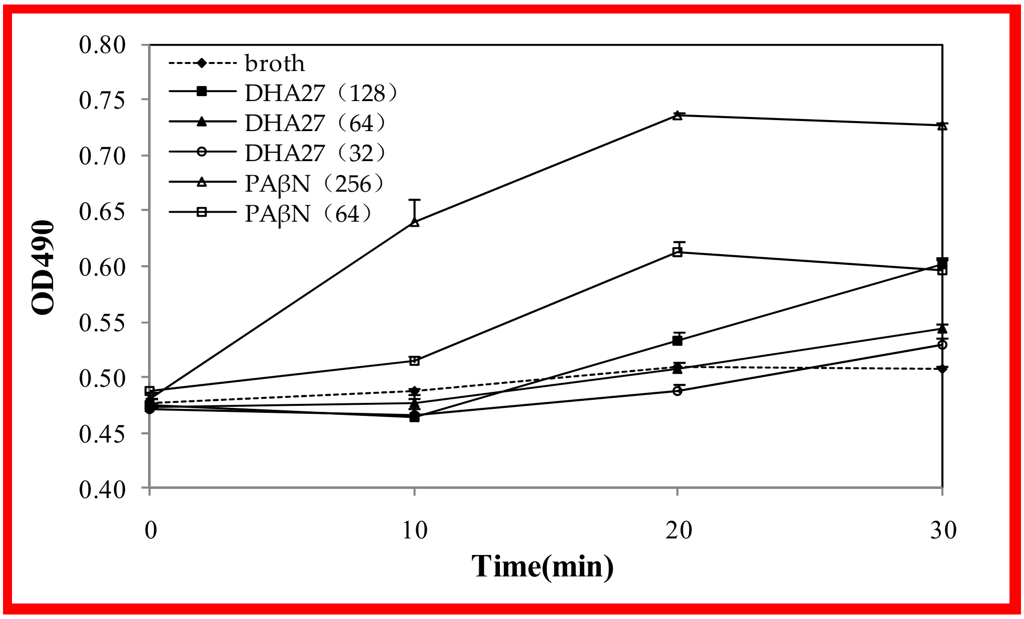

Figure S6. Effect of DHA27 on the permeability of the bacterial membrane within *E. coli* ATCC 35218. *E. coli* ATCC 35218 with constitutive expression of chromosomal β-lactamase were grown in LB broth, harvested, and washed in 50 mmol/L potassium phosphate buffer. The cells were subsequently resuspended in the same buffer to OD_{600} of 0.5. The cell suspension was treated with CCCP (10 μmol/L).And then, different concentrations of DHA27 (32, 64, 128 mg/L) and PAβN (256 mg/L) were added, respectively. Nitrocefin was then added to a final concentration of 32 mg/L. Hydrolysis of nitrocefin was tested by measuring the change of absorbance at 490 nm with a plate reader.

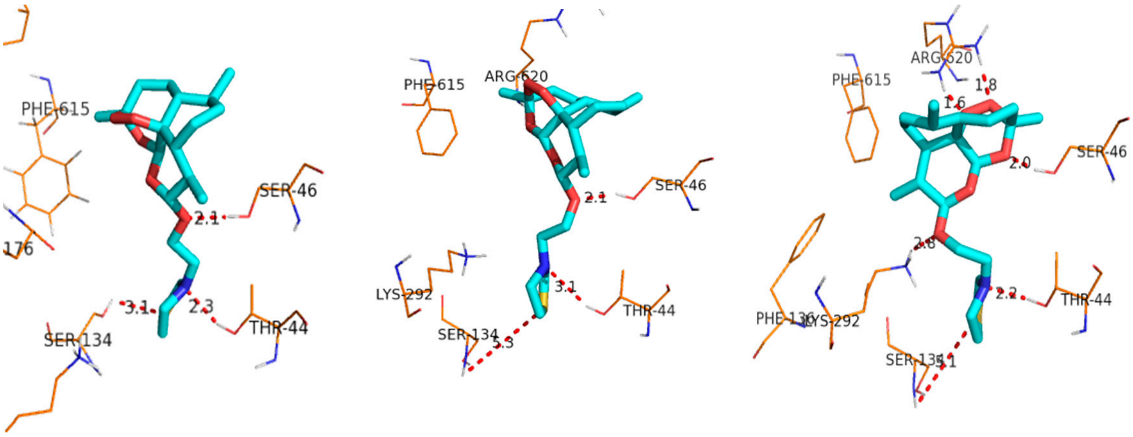

(**A**) (**B**) (**C**)

Template	Donor Atomic	Acceptor Atom	Residues	Mode	Bond Length
2DRD	N	OH	Thr44	H-acc	2.3 Å
	O	OH	Ser46	H-acc	2.1 Å
	S	OH	Ser134	H-acc	3.1 Å
2J8S	N	OH	Thr44	H-acc	2.2 Å
	O	OH	Ser46	H-acc	2.0 Å
	S	OH	Ser134	H-acc	5.1 Å
4DX5	N	OH	Thr44	H-acc	3.1 Å
	O	OH	Ser46	H-acc	2.1 Å
	S	OH	Ser134	H-acc	5.3 Å
	O	NH	Arg620	H-acc	1.8 Å

(**D**)

Figure S7. Molecule docking of DHA27 binding to AcrB. The crystal structure of AcrB (PDB ID 2DRD, 2J8S and 4DX5) were obtained from Protein Data Bank. The molecular docking results were analyzed and represented in the PyMOL 1.3 visualization software. Proteins are shown in gray and green. The compound is shown as thick sticks colored according to the atom type (red, oxygen; cyan, carbon; white, hydrogen; dark blue, nitrogen; yellow, sulfur), and residues are shown in bluish violet and marked. (**A**) Cartoon view of DHA27 docked with AcrB when PDB ID 2DRD was used; (**B**) Cartoon view of DHA27 docked with AcrB when PDB ID 2J8S was used; (**C**) Cartoon view of DHA27 docked with AcrB when PDB ID 4DX5 was used; (D) Details of DHA27 docking with 3 different template of AcrB.

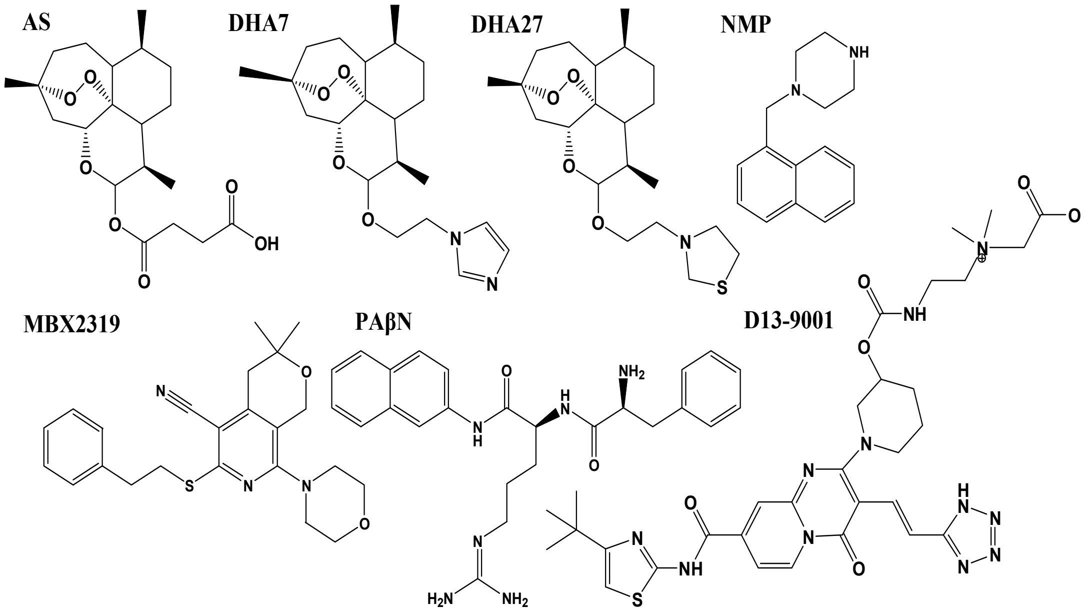

Figure S8. Structures of known AcrB inhibitors.
